# Poly(I:C) Lipoamino Bundle LNPs Induce Tumor Cytotoxicity and Immune Activation with Enhanced Efficacy by Survivin Silencing

**DOI:** 10.3390/ijms27114968

**Published:** 2026-05-30

**Authors:** Mina Yazdi, Zahra Hasheminejad, Khouloud Hachani, Joyce Kache, Melina Grau, Barbara Wollenberg, Ali Bashiri Dezfouli, Ernst Wagner

**Affiliations:** 1 Pharmaceutical Biotechnology, Department of Pharmacy, Ludwig-Maximilians-Universität (LMU), 81377 Munich, Germany; zahrahasheminezhad.khu@gmail.com (Z.H.); melina.grau@cup.uni-muenchen.de (M.G.); 2 CNATM—Cluster for Nucleic Acid Therapeutics Munich, 81377 Munich, Germany; 3 Department of Otolaryngology, Head and Neck Surgery, TUM School of Medicine and Health, Technical University of Munich, 81675 Munich, Germany; khouloud.hachani@tum.de (K.H.); kachejoyce139@gmail.com (J.K.); barbara.wollenberg@tum.de (B.W.); ali.bashiri@tum.de (A.B.D.)

**Keywords:** poly(I:C), lipid nanoparticles, lipoamino fatty acid, cancer, survivin, PBMCs

## Abstract

Synthetic polyinosinic:polycytidylic acid (poly(I:C)) offers an attractive cancer therapeutic by operating on two fronts at once, combining direct tumor cell killing with immunostimulatory activity. Yet, these dual functions can only be efficiently harnessed when intracellular delivery is sufficiently effective to enable poly(I:C) to reach and activate its intracellular receptors. We addressed this delivery challenge by developing pH-responsive formulations using lipoamino fatty acid xenopeptide (LAF-XP) carriers, composed of polar cationizable succinoyl tetraethylene pentamine (Stp) and apolar cationizable LAF building blocks in defined architectures. In particular, poly(I:C)-lipid nanoparticles (LNPs) formulated with bundle LAF_4_-Stp_1_ XP carriers displayed increased anti-tumoral activity at decreased dosage across multiple cancer cell models, compared to control formulations. In parallel, LAF-XP LNP-delivered poly(I:C) activated immune responses, including CXCL10 production by tumor cells, and activation of peripheral blood mononuclear cells (PBMCs), characterized by increased phenotypic markers (CD69 and LAMP-1/CD107a) and functional molecules (e.g., IFN-γ and granzyme B). Conditioned supernatant of pre-stimulated PBMCs with poly(I:C) reduced cancer cell viability, highlighting the contribution of PBMC-released factors to cancer cell death. Of particular novelty is the combination of poly(I:C) with siRNA-mediated survivin knockdown to increase apoptosis in cancer cells using the bundle LAF-XP LNP. Collectively, our findings establish efficient LAF-XP LNPs as a versatile platform that supports multi-layered therapeutic strategies.

## 1. Introduction

Cancer imposes a major burden on the global healthcare system due to the steady increase in both incidence and mortality rates [1]. Despite significant progress, many current therapeutic modalities fail to meet durable clinical outcomes, particularly in patients with advanced or metastatic cancers, driving the need for more effective and safe therapeutic strategies [2,3].

Immunotherapy has been recognized as one of the most promising approaches to fighting cancer. Rather than directly targeting tumor cells, this strategy aims to enhance or restore the natural immune defense mechanisms, while maintaining patient safety [4]. Several key examples include cancer vaccines (e.g., Sipuleucel-T) [5], immune checkpoint inhibitors (e.g., Pembrolizumab) [6], and adoptive cell transfer (e.g., CAR-engineering immune cells) [7,8]. One promising strategy involves activating immune-sensing receptors that initiate and promote immune responses. A key group of such receptors is pattern recognition receptors (PRRs), which detect pathogenic and endogenous danger signals and coordinate immune activation, linking innate and adaptive immunity during infection, tissue damage, and tumorigenesis [9]. Among these receptors are endosomal toll-like receptor 3 (TLR3) and cytosolic retinoic acid-inducible gene I (RIG-I)-like receptors (RLRs), including RIG-I and melanoma differentiation-associated gene 5 (MDA5). Activation of these receptors can trigger anti-tumor immune responses and, in certain contexts, induce tumor cell-intrinsic cytotoxicity; therefore, agonists targeting them are increasingly recognized as potential therapeutic agents [10,11,12].

Poly(I:C), a synthetic double-stranded RNA (dsRNA) analogue, mimics a viral RNA infection within the cells and acts as a potent agonist of TLR3 [13]. Depending on its molecular length, poly(I:C) can also be recognized by cytoplasmic receptors (i.e., MDA5 and RIG-I), thereby engaging multiple RNA-sensing pathways [14]. Through these pathways, poly(I:C) triggers both innate and adaptive immune responses, leading to the upregulation of type I interferons (IFN-I) and proinflammatory cytokines, as well as the activation of NK cells, maturation of DCs, and cross-priming of CD8+ T cells [15]. Aside from its immunostimulatory activity, evidence indicates that poly(I:C) can directly induce cell death in a wide range of cancer cell types [16,17]. Accordingly, poly(I:C) and its derivatives are often evaluated as vaccine adjuvants or in combination with other therapeutic modalities, such as radiotherapy, in several clinical trials, shedding light on the broad applicability of poly(I:C) in cancer treatment [17,18,19,20,21].

Developing lipid- or polymer-based systems to efficiently deliver poly(I:C) to its target receptor in immune or cancer cells is critical for maximizing efficacy in cancer treatment [22,23,24,25,26,27]. Lipid nanoparticles (LNPs) have gained considerable attention as versatile carriers for therapeutic RNA delivery [28]. The FDA approval of the first siRNA-based drug, Onpattro, in 2018, together with the clinical success of the mRNA-based COVID-19 vaccines, further emphasizes the potential of LNP technology [29,30,31]. LNPs are typically composed of ionizable lipids, helper lipids, polyethylene glycol (PEG) lipids, and cholesterol, each contributing to the structural and functional properties of LNPs [32]. Acknowledging the pivotal role of ionizable lipids in RNA encapsulation within LNPs and in endosomal escape via pH-sensitive protonation, their structures can influence the safety profile and delivery efficiency of LNPs to target cells [33,34].

Since the anti-tumor activity of poly(I:C) strongly depends on efficient intracellular transport, our group has previously developed several non-viral delivery strategies for poly(I:C). Our earlier efforts explored targeted polyplex systems, using epidermal growth factor (EGF)-conjugated PEI for targeting EGF receptor (EGFR)-overexpressing tumors [24,25] or methotrexate (MTX)-functionalized, succinoyl tetraethylene pentamine (Stp)-based polyplexes for folate receptor targeting [35], both of which demonstrated promising tumor-selective activity. We recently reported double pH-responsive lipoamino fatty acid (LAF)-Stp xenopeptide (XP) carriers [36,37,38] with compelling performance both in polyplex [36,37,38,39] and LNP [40,41] formulations. In the current work, we focused on LNP formulations based on the LAF_4_-Stp_1_ XPs for the delivery of poly(I:C). These formulations triggered the desired functional outcomes in target cells, including both cancer and immune cells of the blood, resulting in enhanced anti-tumor effects. The LNP system opened the possibility to deliver a novel combination of poly(I:C) and anti-tumoral survivin-targeting siRNA, driven by the high transfection efficiency of the applied LAF-XP carrier. This allows the use of lower effective doses, which could later translate into improved, safer therapeutic outcomes.

## 2. Results and Discussion

### 2.1. Poly(I:C)-LNP Formulation Using Ionizable LAF-XP Carriers

**Scheme 1:**
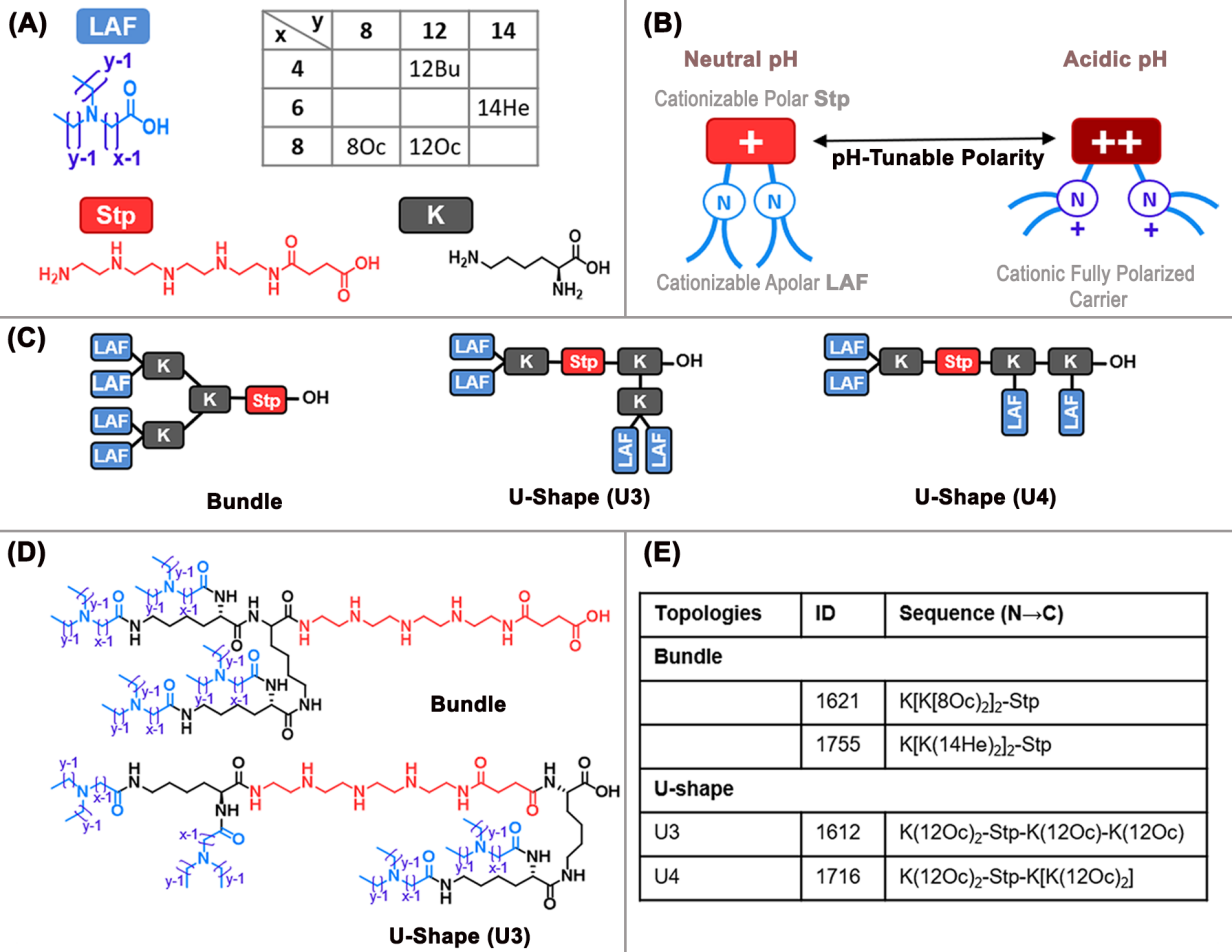
Lipoamino fatty acid (LAF) xenopeptide (XP) nanocarriers for LNP-mediated poly(I:C) delivery. (**A**) Modular design and synthesis strategy of XPs. The polar succinoyl tetraethylene pentamine (Stp) and apolar LAF building blocks were covalently connected via lysine (K) branching unit. Regarding the structural annotation of LAF motifs, the digits (8, 12, 14) and two letters (Oc, Bu, He) indicate the C-atom number of terminal alkyl chains and amino fatty acid type, respectively. Oc, 8-aminooctanoic acid; He, 6-aminohexanoic acid; Bu, 4-aminobutanoic acid. (**B**) Protonation-driven shift in the physicochemical character of the LAF unit, transitioning from an apolar state at neutral pH to a more polar, protonated form under acidic pH. (**C**) Schematic illustration of bundle and U-shape topologies, each constructed at a LAF:Stp ratio of 4:1 (LAF_4_:Stp_1_). (**D**) Representative chemical structure of bundle and U-shape carriers. (**E**) Selected LAF-XP candidates with bundle and U-shape topologies for LNP formulation.

The presence of ionizable lipids in LNP formulations is indispensable for improving encapsulation efficiency and intracellular cargo release, owing to their pH-responsive protonation characteristics [42]. During nanoparticle assembly under acidic conditions, their protonation enables electrostatic complexation with negatively charged nucleic acids, and they remain largely neutral at physiological pH. Following cellular uptake, the progressive acidification of endosomal compartments triggers the reprotonation of ionizable lipids, which promotes endosomal membrane destabilization and cytosolic release of the cargo. In addition to controlling transfection efficiency, ionizable lipids can influence pharmacokinetics, safety profile, and interactions of LNPs with specific target tissues and cells, motivating extensive efforts to design novel ionizable lipids for next-generation LNP formulations [43,44,45,46,47,48,49]. Aligned with this objective, a library of carriers was previously constructed by integrating polar, cationizable Stp and apolar LAF motifs, in which lysine acts as a branching point to covalently connect LAFs to Stp (Scheme 1A) [36,37]. Novel LAF building blocks, incorporating a tertiary amine centered within a tri-alkyl hydrophobic framework, were synthesized via reductive amination of amino fatty acids with fatty aldehydes of different chain lengths. For structural annotation, the alkyl chain lengths are encoded numerically (8, 12, 14), while the amino fatty acid types are indicated by two-letter abbreviations (Oc, Bu, He) (Scheme 1A). Functionally, LAF-XP carriers have double pH-responsive behavior, arising from the tertiary amine in the LAF, together with the secondary aminoethylenes of Stp [36,37]. Moreover, LAFs confer a protonation-dependent polarity change to the hydrophobic moieties of XP carriers under acidic pH (Scheme 1B).

These sequence-defined nanocarriers were assembled by solid-phase peptide synthesis (SPPS). By adjusting motif ratios and spatial arrangement, various structural topologies, such as bundle (hydrophilic/lipophilic blocks) and U-shapes (lipophilic ends), were generated (Scheme 1C,D). Systematic modulation of LAF type, LAF/Stp ratio, and carrier topology enabled the identification of top-performing XP carriers with superior delivery efficiency for mRNA, plasmid DNA (pDNA), and siRNA in the form of polyplexes [36]. In particular, the more lipophilic LAF_4_-Stp_1_ XPs, depending on their bundle or U-shape topologies, have proven to be very effective in LNP formulations for delivering mRNA or siRNA both in vitro and in vivo [40,41]. These findings prompted further investigation into the application of top-performing LAF-XP LNPs for poly(I:C) delivery. Individual carriers were specified by a four-digit identification (ID) number corresponding to the internal library code. In the present study, four distinct LAF-XP carriers (bundles 1621 and 1755, and U-shapes 1612 and 1716) were applied for LNP formulation, with their chemical structures and corresponding ID numbers detailed in Scheme 1E. LNPs were prepared from cholesterol, 1,2-distearoyl-sn-glycero-3-phosphocholine (DSPC), 1,2-dimyristoyl-rac-glycero-3-methoxypolyethylene glycol-2000 (DMG-PEG2000), and an ionizable LAF-XP carrier. Although much research has focused on modifying ionizable lipid structures to improve delivery efficiency, other LNP components, including PEG-lipids, cholesterol, and phospholipids, also play important roles in stability, biodistribution, and delivery performance, and therefore require careful optimization as well [32]. Herein, the LNP components were combined at an established, optimized molar ratio, as described in earlier works [40,41]. LNP formulations containing the clinically validated ionizable lipids DLin-MC3-DMA (MC3) and SM102 were included as reference systems for benchmarking purposes. For clarity, LNPs containing MC3 and SM102 are referred to as SM102-LNP and MC3-LNP, respectively, and LNPs prepared with LAF_4_-Stp_1_ XP carriers are denoted as XP-LNPs or by their carrier ID number (e.g., 1621-LNP).

The poly(I:C)-LNP formulations were then characterized for their physicochemical properties prior to biological evaluation. Hydrodynamic diameter (z-average), polydispersity index (PDI), and zeta potential were determined by dynamic and electrophoretic light scattering (DLS and ELS), and encapsulation efficiency (EE) was quantified to assess cargo loading (Figure S1). LNPs containing single-stranded RNA poly(I) were included for comparison.

The SM102- and MC3-LNPs exhibited uniform particle sizes of approximately 150 nm (Figure S1A,B). Among the XP-LNPs, 1755, 1612, and 1716 yielded LNPs within an acceptable size range (≈150–200 nm) with slightly broader size distribution than reference LNPs. As opposed to the others, 1621-LNP tended to form larger particles (≈300 nm). The PDI difference between poly(I)- and poly(I:C)-LNPs was low, indicating that cargo type had no significant impact on nanoparticle uniformity. Zeta potential measurements demonstrated near-neutral to slightly negative surface charges (Figure S1C). The EE was consistently high across all LNP formulations, exceeding 75% (Figure S1D). When set side by side, XP-LNPs achieved robust cargo encapsulation, comparable to, and in some instances (1755, 1612, and 1716) modestly higher than the reference LNPs. Transmission electron microscopy (TEM) imaging revealed XP-LNPs with predominantly spherical to near-spherical morphology, supporting the successful formation and morphological integrity of the formulations (Figure S1E).

For the aqueous and serum stability assessment, LNPs were incubated in formulation buffer in the absence or presence of 45% (*v*/*v*) fetal bovine serum (FBS) for 2 h at 37 °C (Figure S2A). Compared with the free migration of non-encapsulated poly(I:C), LAF-XP and reference LNPs showed efficient poly(I:C) entrapment. Even in the presence of serum, a substantial fraction of poly(I:C) remained retained within the formulations, except for a slight release for SM102-LNP. Furthermore, the storage stability of formulations was assessed by monitoring their particle size and PDI over 6 days at 4 °C (Figure S2B). The parameters remained largely unchanged over this period, indicating that the formulations maintained their physicochemical stability under these storage conditions. Overall, the XP-LNP formulations exhibited physicochemical properties that favor effective cellular interactions and were therefore applied for further evaluation.

### 2.2. In Vitro Anti-Tumor Activity of Poly(I:C) Encapsulated in XP-LNPs

As reported in numerous studies, poly(I:C) exerts therapeutic activity against a wide range of cancer types such as breast [50], prostate [51,52], colon [53], lung [54], head and neck [55], gastric cancer [56], renal cell carcinoma [57], hepatocellular carcinoma [58,59], glioblastoma [60], and neuroblastoma [61]. Its anti-cancer activity is associated with reduced cellular proliferation and the induction of apoptotic cell death, mediated through TLR-3 activation [51,53,59], although in some cancers cytosolic RLRs may predominate, while in others both TLR-3 and RLR are involved, depending on the cancer cell type [50,55,56,57,58,60,61]. TLR3 has been reported to exert both apoptotic and tumor-promoting effects in certain cancer cell lines [62,63], which may, in part, relate to differences in receptor localization and downstream signaling context. However, efficient intracellular delivery of poly(I:C), enabling engagement of endosomal and cytosolic RNA-sensing receptors, can lead to the anti-tumoral outcome.

**Figure 1:**
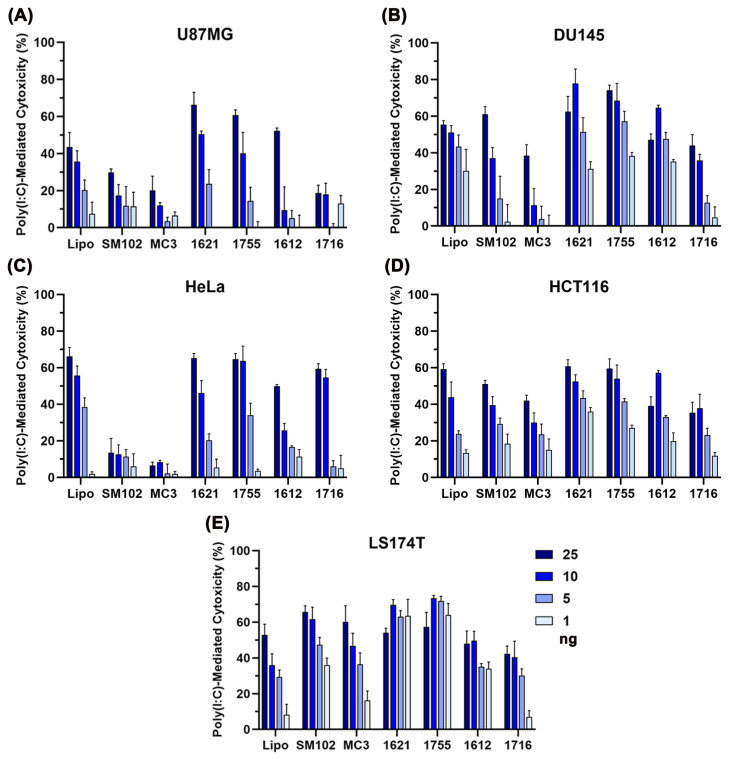
Cytotoxic activity of XP-LNP-encapsulated poly(I:C) in multiple human cancer cell lines. LNPs were formulated as described in the Experimental Section. Lipofectamine (Lipo), SM102-, and MC3-LNPs served as reference controls. Cancer cells (4000 cells per well containing 100 µL culture medium), including (**A**) glioblastoma U87MG, (**B**) prostate DU145, (**C**) cervical adenocarcinoma HeLa, and colorectal carcinoma cell lines (**D**) HCT116 and (**E**) LS174T were treated with different doses of poly(I:C) (25, 10, 5, and 1 ng per well). Cell viability was assessed using the MTT assay and is expressed as a percentage relative to buffer-treated cells (mean + SD, *n* = 3). Poly(I:C)-mediated cytotoxicity was calculated as the difference between the viability values obtained for poly(I)-treated and poly(I:C)-treated samples.

To determine whether XP-LNPs efficiently deliver poly(I:C) into cancer cells and elicit cytotoxicity, we assessed cell viability after treatment with poly(I:C)-loaded formulations at various doses (25, 10, 5, and 1 ng per 4000 cells/well) for 48 h using the MTT assay. XP-LNPs were evaluated alongside Lipofectamine as well as reference SM102- and MC3-LNPs. Experiments were conducted in multiple cancer cell lines, including glioblastoma (U87MG), prostate (DU145), cervical (HeLa), and colorectal cancer cells (HCT116 and LS174T). Poly(I)-loaded formulations were tested in parallel to distinguish poly(I:C)-induced cytotoxicity from potential carrier-related and non-specific RNA effects. Poly(I), which lacks the double-stranded structure required for TLR3 and RLR activation, can serve as a control to account for these non-specific effects. The corresponding total viability data for poly(I:C)- and poly(I)-loaded formulations are provided in Figures S3–S8. To better interpret the findings, the specific effect of poly(I:C) was determined by subtracting the viability values obtained with poly(I:C) from those measured after poly(I) treatment. This difference was defined as poly(I:C)-mediated cytotoxicity (%) (Figure 1).

In U87MG glioblastoma cells, the poly(I:C)-mediated cytotoxicity increased in a dose-dependent manner for most formulations (Figure 1A). At the highest tested dose (25 ng), the strongest poly(I:C) effect was observed with 1621-LNP (~65%), followed by 1755- and 1612-LNPs, which showed higher efficacy than previously reported delivery strategies [24,25,35]. Lipofectamine at 25 ng resulted in ~40% cell killing, comparable to bundle XP-LNPs at 10 ng, whereas MC3- and SM102-LNPs showed substantially lower effects at the same doses. The 1716-LNP induced low cytotoxicity and relatively high non-specific effects (Figure S3). At 10 ng poly(I:C), the ranking of formulations was similar, albeit with a reduced effect size. At the lowest dose value (5 and 1 ng), cytotoxicity remained below 30% for all platforms. Interestingly, in the mouse brain-derived tumor cell model N2A (neuroblastoma), poly(I:C) treatment resulted in minimal cytotoxicity (Figure S4). Even at higher doses (25 and 10 ng), cell killing was low and detectable only with bundle XP-LNPs.

An almost similar dose-dependent trend was detected in DU145 prostate cancer cells, which exhibited greater sensitivity to poly(I:C) than U87MG (Figure 1B vs. Figure 1A). Bundle 1621- and 1755-LNPs maintained a favorable activity profile throughout the tested dose range; however, 1621-LNP showed slight non-specific effects at 25 ng (Figure S5). In contrast, the U-shape 1612- and 1716-LNPs caused higher non-specific effects (particularly at 25 ng), and their overall poly(I:C)-mediated cytotoxicity was lower than that of the bundle XP-LNPs. SM102, followed by MC3-LNP, displayed moderate activity, mainly at higher doses (25 and 10 ng). Lipofectamine also induced notable poly(I:C)-mediated cell killing, still with efficacy below that achieved with the bundle XP-LNPs. Although experimental conditions vary between studies, the poly(I:C) effect obtained with XP-LNPs in DU145 cells exceeds previously reported outcomes with free poly(I:C) or poly(I:C) delivered using conventional transfection approaches [52].

In HeLa cells, the poly(I:C) effect showed a dose-dependent pattern with XP-LNPs and Lipofectamine (Figure 1C). Among the XP-LNPs, 1755-LNP reached the highest activity, approaching the levels similar to those of Lipofectamine, followed by 1621-LNP and the U-shape 1716- and 1612-LNPs. Unlike the trends observed in U87MG and DU145 cells, MC3- and SM102-LNPs led to only limited cell killing in HeLa cells (<20%), even at the highest poly(I:C) dose. In this cell model, 1716- demonstrated higher efficiency than 1612-LNP. At a lower dose of 5 ng, no significant loss of viability was found, except for 1755-LNP and Lipofectamine, which still induced ~40% cytotoxicity. The non-specific effect of XP-LNPs was generally low in HeLa cells (Figure S6).

The poly(I:C)-mediated cytotoxic effect was further evaluated in the colorectal cancer cell lines HCT116 and LS174T (Figure 1D,E), which differ in their biological characteristics [64]. In HCT116 cells, the bundle XP-LNPs revealed a dose-dependent activity profile in line with that of MC3- and SM102-LNPs. Lipofectamine and the U-shape XP-LNPs exhibited lower poly(I:C)-based cytotoxicity. Differences between formulations became less pronounced at 25 and 10 ng, where the formulations (except the U-shape) reached similar efficacy levels. In LS174T cells, a clearer distinction between formulations was seen. Bundle XP-LNPs induced remarkable cytotoxicity, even at 1 ng poly(I:C). Lipofectamine, SM102-, and MC3-LNPs reached comparable levels only at the highest dose (25 ng), with decreasing effects at lower doses. The U-shape XP-LNPs were less efficient at delivering poly(I:C) than the bundle XP-LNPs. Non-specific effects were noticeable for the U-shape in comparison to bundle-based LNPs (especially at 25 ng) and were more evident in LS174T than in HCT116 cells (Figures S7 and S8).

Taken together, the effect of poly(I:C) on cell viability showed a clear dependence on the cellular model and the delivery platform. Among the tested formulations, bundle 1621- and 1755-LNPs generally produced the strongest responses in the majority of cell lines. This is particularly evident in colorectal carcinoma LS174T cells, where these formulations induced substantial cytotoxicity already at 1 ng poly(I:C), whereas Lipofectamine and reference LNPs (SM102- and MC3-LNPs) required up to 25-fold higher doses to achieve comparable effects. Moreover, bundle XP-LNPs induced ~70% cytotoxicity in U87MG cells at 25 ng poly(I:C), approximately two-fold higher than that of the reference LNPs at a similar dose, and led to even greater relative poly(I:C) activity in HeLa cells, reaching up to six-fold higher poly(I:C)-mediated cytotoxicity.

It is worth discussing the performance of LAF-XP carriers for poly(I:C) delivery as polyplexes. To this end, candidates were selected from previously published top performers, including the U-shaped carriers 1611 (LAF_2_-Stp_1_) and 1719 (LAF_4_-Stp_2_), and the bundle carrier 1752 (LAF_4_-Stp_1_) [36] (Figure S9A). The polyplexes were formulated at carrier-specific optimized N/P ratios, selected based on previous optimization of related LAF-XP nucleic acid polyplexes [36] and further assessed for poly(I:C) delivery by physicochemical characterization and biological activity assessment. DLS and ELS revealed the formation of positively charged nanoparticles with hydrodynamic diameters of approximately 100–150 nm, with a more homogeneous distribution for 1719 and 1752 than for 1611 (Figure S9B–D). The agarose gel shift analysis confirmed efficient and stable poly(I:C) complexation and retention within the polyplexes at the selected N/P ratios (Figure S9E). In U87MG cells, poly(I:C)-mediated delivery by 1719 polyplexes achieved ~90% reduction in cell viability at the dose of 25 ng (Figure S10A). Previously reported LPEI-mediated delivery at a similar dose resulted in ~50% reduction in cell viability and showed a rapid increase in non-specific cytotoxicity at higher doses [25]. In comparison, LAF-XP polyplexes 1611 and 1752 showed favorable in vitro anti-tumor activity profiles, with clear dose-dependent effects starting at around 10 ng/well and minimal non-specific cytotoxicity at a higher dose of 100 ng/well in U87MG. In HeLa cells, LPEI polyplexes induced less than 20% cell killing even at a high dose of 100 ng poly(I:C). In contrast, 1611 and 1752 achieved ~50% decrease in cell viability at a low poly(I:C) dose of 5 ng, and 1719 approached ~80% at 25 ng (Figure S10B), further underscoring the superior efficiency of LAF-XP-based formulations for poly(I:C) delivery over standard LPEI polyplexes.

### 2.3. XP-LNP-Mediated Poly(I:C) Delivery Induces CXCL10 Production in Tumor Cells

Despite the clinical success of immunotherapy, its efficacy in many solid tumors is hindered by multiple barriers, including intrinsic and acquired resistance mechanisms, as well as insufficient immune cell infiltration into the tumor microenvironment (TME) [65]. The recruitment of immune cells into tumor tissue is tightly regulated by cytokine-chemokine networks, which play a central role in shaping anti-tumor immunity [66]. CXCL10, also known as IFN gamma-induced protein 10 (IP-10), is a key IFN-inducible chemokine involved in inflammation and immune surveillance. It contributes to anti-tumor immune response by facilitating the recruitment of effector cells, such as cytotoxic T lymphocytes and natural killer cells, into the TME [67].

**Figure 2:**
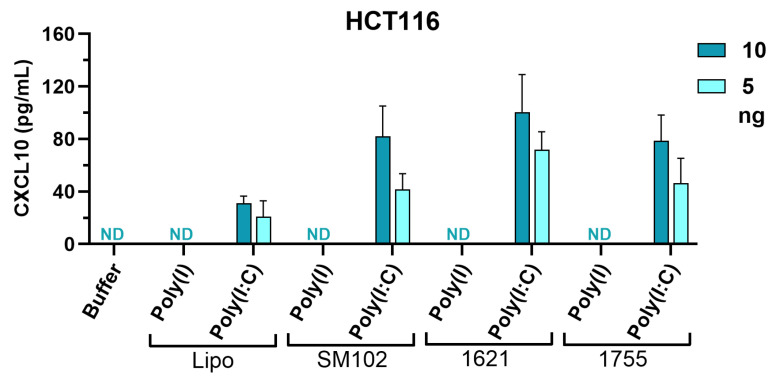
CXCL10 secretion by HCT116 cells after exposure to poly(I:C) using XP-LNPs. LNPs were formulated as described in the Experimental Section. Lipo and SM102-LNP served as controls and were compared with bundle XP-LNPs (1621- and 1755-LNPs). HCT116 cells (12,000 cells per well) were treated at two poly(I:C) doses (10 and 5 ng per 4000 cells). In parallel, poly(I)-loaded formulations were used to account for non-specific effects. After 24 h, supernatants were collected, and CXCL10 levels were quantified using ELISA. The data are expressed as the mean + SD from two independent experiments, each performed in duplicate.

Poly(I:C) and its derivatives can induce chemokine production, including CXCL10, offering an additional therapeutic advantage beyond direct tumor cell killing [68,69,70,71]. To assess CXCL10 production following poly(I:C) treatment, CXCL10 levels were measured in cancer cell culture supernatants by ELISA. The cells were transfected with Lipofectamine and SM102-, 1621-, and 1755-LNPs, selected for their high activity in HCT116 cells, at two doses (10 and 5 ng per 4000 cells) for 24 h. As depicted in Figure 2, CXCL10 was not detectable in the buffer and poly(I)-treated groups. In contrast, the SM102, 1621, and 1755 formulations, as well as Lipofectamine, induced measurable CXCL10 production in HCT116, with a dose-dependent trend. Among these, 1621-LNP caused greater CXCL10 secretion. Consistent with this, previous studies have demonstrated that poly(I:C)-induced TLR3 signaling triggers robust CXCL10 production in esophageal squamous cell carcinoma, leading to both immune cell infiltration and apoptosis induction [71].

### 2.4. Enhanced Anti-Tumor Immune Activity of Poly(I:C) in Peripheral Blood Mononuclear Cells (PBMCs) Enabled by XP-LNP Delivery

Ex vivo stimulation of PBMCs is a valuable model to study the inflammatory and immunomodulatory effects of poly(I:C) beyond its direct action on tumor cells [72]. Owing to their heterogeneous composition, they provide a more comprehensive view of immune activation than single-cell-type systems [72].

**Figure 3:**
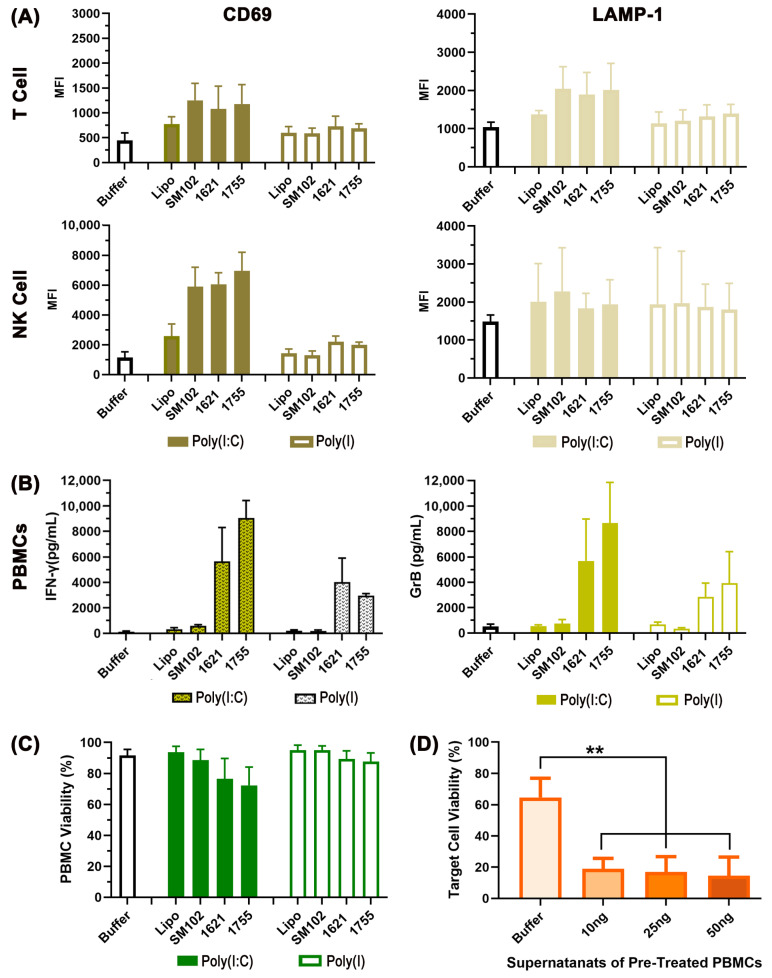
Immunostimulatory effect of XP-LNP-encapsulated poly(I:C) on peripheral blood mononuclear cells (PBMCs). LNPs were formulated as described in the Experimental Section. Lipo and SM102-LNP served as controls and were compared with bundle XP-LNPs (1621- and 1755-LNPs). Corresponding formulations with poly(I) were included to control non-specific effects. Healthy-donor isolated PBMCs (4 × 10^5^ cells) were treated with 50 ng poly(I:C) for 24 h prior to the following analysis. (**A**) Surface activation marker CD69 and degranulation marker LAMP-1 expression measured in T cells (upper panels) and NK cells (lower panels) using flow cytometry. Mean fluorescence intensity (MFI) is reported as the mean + SD from three independent donors (*n* = 3), each in duplicate. (**B**) IFN-γ and Granzyme B (GrB) quantified in PBMC culture supernatants using ELISA. The data are expressed as the mean + SD from four independent donors (*n* = 4), each in duplicate. (**C**) PBMC viability assessed by flow cytometry and defined as the percentage of SYTOX^TM^ Blue-negative cells (mean + SD, from two independent donors (*n* = 2), each in duplicate). (**D**) Anti-tumor activity of conditioned supernatants derived from PBMCs pretreated with 1755-LNP/poly(I:C) at indicated doses (50, 25, and 10 ng) for 24 h. Supernatants were added to the wells containing LS174T cancer cells. The ratio of the involved cells was PBMC to target cancer cells of 4 to 1. After 24 h, cell viability was assessed using flow cytometry with SYTOX Blue staining (mean + SD, from two independent experiments (*n* = 2), each in duplicate). Statistical significance is indicated as ** *p* < 0.01.

Given the use of poly(I:C) as an adjuvant in cancer therapy and vaccination, we aimed to investigate the potential of bundle XP-LNPs (1755 and 1621) to induce anti-tumoral immune responses in PBMCs via poly(I:C) delivery, in comparison to SM102-LNP and Lipofectamine. PBMCs were exposed to the respective formulations for 24 h, and the effect of poly(I:C) was monitored through CD69 expression, an early indicator of cellular activation, in T cells and NK cells, which can respond directly or indirectly to poly(I:C) [73,74] (gating strategy shown in Figure S11). LNP-delivered poly(I:C) upregulated CD69 on NK cells and, to a lesser extent, on T cells, with similar trends observed for SM102- and XP-LNPs (Figure 3A). Even at lower poly(I:C) doses, 1755-LNP maintained detectable CD69 upregulation on both NK and T cell populations (Figure S12). Lipofectamine was less efficient than LNP formulations in inducing CD69 expression.

Since CD69 alone does not necessarily reflect effector cytotoxic function, degranulation was additionally evaluated by assessing surface LAMP-1 (CD107a) expression (gating strategy shown in Figure S11). In NK cells, both poly(I)- and poly(I:C)-loaded formulations led to almost similar LAMP-1 expression, limiting a definitive interpretation of NK cell activation beyond CD69 (Figure 3A). This observation is consistent with previous reports indicating that LAMP-1 expression does not always correlate with other functional markers [75]. A different pattern emerged in T cells, with LAMP-1 elevation restricted to poly(I:C), that supports a clearer association of CD69 and LAMP-1 with T cell activation. Of note, we should consider that poly(I:C)-mediated activation of T cells is largely indirect and occurs through bystander signaling within PBMCs rather than direct poly(I:C) sensing [76,77]. To mention, CD69 and LAMP-1 were analyzed in NK and T cells rather than the whole PBMC population because these populations are central effectors of anti-tumor immunity through direct cytotoxicity and release of soluble mediators such as cytokines.

To extend this analysis beyond surface activation markers and evaluate functional output at the whole PBMC population, Granzyme B (GrB) and IFN-γ levels were measured following poly(I:C) treatment. Enhanced GrB secretion and increased pro-inflammatory IFN-γ production indicate functional effector responses induced by poly(I:C) (Figure 3B), as reported previously [73,78,79]. XP-LNPs triggered markedly higher IFN-γ and GrB production compared to SM102-LNP and Lipofectamine, and 1755-LNP moderately outperformed 1621-LNP. In contrast, the poly(I)-loaded XP-LNPs led to lower IFN-γ and GrB levels, which implies the specificity of the response to poly(I:C). To ensure that the induced immune activation was not driven by cytotoxic effects, PBMC viability was also assessed (Figure 3C). Across most formulations, viability exceeded 80%, with only a slight reduction detected for bundle XP-LNP/poly(I:C). As LNPs encapsulating poly(I) did not affect viability to the same extent, this decrease can be attributed to the poly(I:C) cargo rather than the delivery platform. The gating strategy for this cell viability evaluation is shown in Figure S13.

Whether PBMC activation can translate into anti-tumor activity was investigated by applying conditioned supernatants from PBMCs pre-stimulated with 1755-LNP at different poly(I:C) doses (50, 25, and 10 ng) to LS174T cancer cells for 24 h (Figure 3D). LS174T cells were selected as representative target cancer cells due to their pronounced sensitivity to 1755-LNP-mediated poly(I:C) delivery, as shown by their strong response even at low poly(I:C) doses. These supernatants induced significant reductions in cancer cell viability, in contrast to the limited effects caused by supernatants from unstimulated PBMCs. This effect can be attributed, at least in part, to PBMC-derived effectors, such as IFN-γ and GrB, which mediate cytotoxicity and modulate tumor cell susceptibility [80]. It is important to point out that the conditioned supernatants likely contain both effector molecules and residual active poly(I:C)-LNPs remaining in the culture medium. This setup creates a clinically relevant scenario in which tumor cells are simultaneously exposed to the combined effects of direct poly(I:C)-mediated cytotoxicity and PBMC-derived effectors. Although the individual contributions of each component cannot be fully distinguished, their combined action may amplify the overall therapeutic efficacy within TME. For instance, IFN-γ has been shown to increase tumor cell susceptibility to apoptosis and to synergize with poly(I:C) to reduce tumor cell viability by coordinating cell cycle arrest and apoptotic pathways [80]. As such, our results underscore the functional contribution of poly(I:C)-triggered PBMC activation to anti-tumor activity. Since the overall poly(I:C)-induced immune activity reflects an interconnected network of diverse immune mediators that may be shaped by dose, formulation, and in vivo biodistribution, future studies encompassing comprehensive cytokine profiling will be important for optimizing this response, defining the therapeutic window, and evaluating potential safety margins.

### 2.5. XP-LNP-Mediated Delivery of Poly(I:C) and Survivin siRNA Promotes Tumor Cell Killing

In the clinical context, using poly(I:C) at the lowest effective dose is particularly important, as higher doses may increase the risk of unwanted inflammatory or off-target effects [81]. In addition, tumor cells often gain resistance to single-agent therapies, and combination approaches targeting distinct or overlapping mechanisms are widely used to improve therapeutic efficacy [82].

Survivin (BIRC5), a member of the inhibitor of apoptosis protein (IAP) family, is highly expressed in many cancers, but is low or absent in most normal tissues [83]. Elevated survivin levels in tumor cells, such as colorectal cancer, have been associated with carcinogenesis, poor clinical prognosis, therapy resistance, and reduced patient survival [84]. Given its role in regulating apoptosis and cell survival, survivin has been a promising target for anti-cancer therapy [83,85,86,87].

To examine the impact of survivin depletion on the response to poly(I:C), experiments were performed in HCT116 cells using 1755-LNP, a formulation previously identified as an efficient XP-LNP system also for siRNA delivery [41]. This formulation, encapsulating survivin-targeting siRNA (siSurvivin), induced strong survivin knockdown in HCT116 cells, with more than 90% silencing observed at siRNA doses of 25 and 10 ng (per 4000 cells) (Figure S14A). Survivin silencing alone was associated with a maximum 40% reduction in cell viability at the dose of 10 ng siRNA after 72 h. However, increasing the siRNA dose did not further reduce cell viability, likely due to increased non-specific effects at higher doses and/or saturation of the RNA interference (RNAi) response once survivin knockdown was already maximal (Figure S14B).

**Figure 4:**
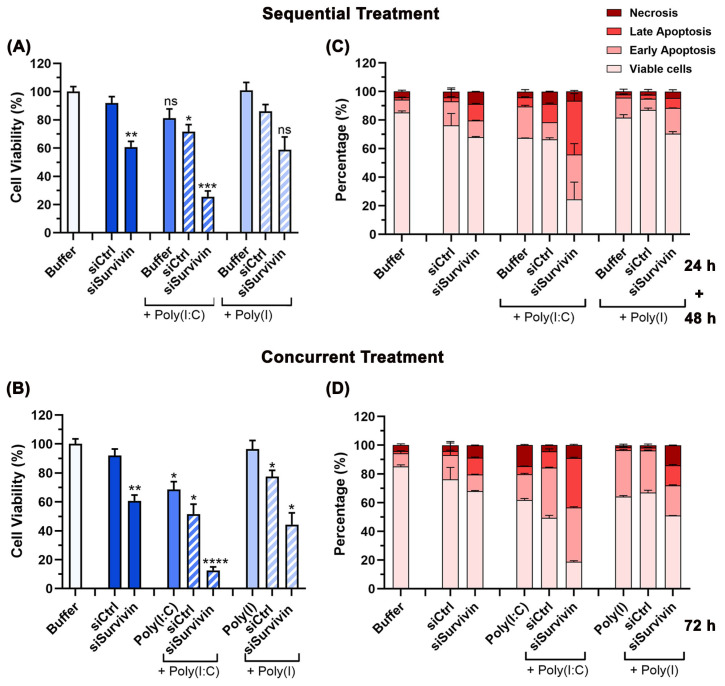
Effect of survivin knockdown and poly(I:C) on the viability and apoptosis induction in HCT116 cells. 1755-LNPs were formulated as described in the *Experimental Section*. For sequential treatment, HCT116 cells were first treated with 1755-LNP encapsulating survivin-targeting siRNA (siSurvivin, 5 ng per 4000 cells) for 24 h, followed by poly(I:C)-LNP (1 ng per 4000 cells) for an additional 48 h. For concurrent treatment, cells were co-treated with siSurvivin-LNP (5 ng) and poly(I:C)-LNP (1 ng) for 72 h. Corresponding formulations loaded with control siRNA (siCtrl) or poly(I) were included to exclude non-specific effects. Cell viability was assessed by MTT assay following (**A**) sequential and (**B**) concurrent treatment and expressed as a percentage relative to buffer-treated cells (mean + SD, *n* = 3). Apoptosis was quantified by Annexin V-FITC/propidium iodide (PI) staining using flow cytometry after (**C**) sequential and (**D**) concurrent treatment strategies. The percentages of viable, early apoptotic, late apoptotic, and necrotic cells are presented as mean + SD (*n* = 2). Statistical significance is indicated as ns = not significant, * *p* ≤ 0.05, ** *p* ≤ 0.01, *** *p* ≤ 0.001, and **** *p* ≤ 0.0001.

Preliminary studies were performed to establish the optimal dose for treatments and appropriate time points for cell viability measurements. Based on these results, 5 ng siSurvivin and 1 ng poly(I:C) were selected for subsequent experiments. Cells were first transfected with siSurvivin-LNP (5 ng per 4000 cells) for 24 h, followed by poly(I:C)-LNP (1 ng per 4000 cells) for an additional 48 h before cell viability assessment (Figure 4A,C). For single-treatment, cells were transfected with either siSurvivin for 72 h or buffer (for 24 h) + poly(I:C) (for an additional 48 h). Survivin silencing reduced cell viability by ~25% compared to control siRNA (siCtrl)-treated cells, and poly(I:C)-mediated cytotoxicity, calculated relative to poly(I), corresponded to ~20% cell death. (Figure 4A). Notably, the sequential combination of siSurvivin and poly(I:C) promoted cell death by approximately 40%, relative to the corresponding siCtrl + poly(I:C) treatment. The specificity of these effects was confirmed by using poly(I)-LNPs after siRNA treatment, which did not produce combinatorial effects.

In agreement with previous reports on cancer cell sensitization through survivin suppression [88,89], survivin knockdown is expected to lower the apoptotic threshold and prime cancer cells for subsequent treatment with a second agent, such as poly(I:C). We also examined whether the treatment sequence is critical for the observed effect. Interestingly, MTT assays showed that concurrent exposure of cells to both siSurvivin-LNP (5 ng) and poly(I:C)-LNP (1 ng) was also significantly more effective than either cargo alone (Figure 4B), suggesting that the enhanced cell death is not strictly dependent on a defined sequential treatment schedule. Rather, survivin silencing and poly(I:C)-triggered death signaling likely act in parallel within the treatment window, despite their different kinetics. Since both siSurvivin and poly(I:C) are known to modulate apoptotic pathways, Annexin V/PI staining was performed to assess apoptosis. As shown in Figure 4C,D, co-treatment markedly increased apoptotic cell death, as assessed using the gating strategy in Figure S15. Efficient silencing of survivin was also confirmed in HeLa cells, accompanied by a modest reduction in cell viability compared to siCtrl-treated cells (Figure S16). HeLa cells exhibited response patterns comparable to HCT116 cells under both sequential and concurrent combination treatments (Figure S17).

Overall, our study introduces, for the first time, a promising strategy that combines LNP-mediated delivery of survivin-targeting siRNA with poly(I:C), applied either sequentially or concurrently, to enhance anticancer activity without the need for dose escalation of each cargo. Poly(I:C) provides a distinct therapeutic advantage in our combination strategy, as it not only induces tumor cell death but also activates immune signaling pathways, thereby integrating multiple anti-cancer mechanisms into a single treatment strategy.

## 3. Materials and Methods

### 3.1. Materials

Cell culture consumables were purchased from Faust Lab Sciences (Klettgau, Germany). McCoy’s 5A, RPMI-1640, DMEM low glucose (1 g/L glucose), FBS, antibiotics (penicillin, streptomycin), and trypsin/EDTA were purchased from Sigma Aldrich (Munich, Germany) and PAN-Biotech (Aidenbach, Germany). All the cell culture flasks and pipettes were bought from Sarstedt (Nümbrecht, Germany). HEPES was purchased from Biomol GmbH (Hamburg, Germany). Disodium ethylenediaminetetraacetic acid (EDTA), poly(I:C), and poly(I) were purchased from Merck (Darmstadt, Germany). Further solvents and reagents were obtained from Sigma-Aldrich (Munich, Germany), Iris Biotech (Marktredwitz, Germany), Merck (Darmstadt, Germany), or AppliChem (Darmstadt, Germany). Cholesterol was purchased from Sigma-Aldrich (Munich, Germany), and DSPC and DMG-PEG2000 from Avanti Polar Lipids (Alabaster, AL, USA). The ionizable lipid DLin-MC3-DMA was purchased from MedChemExpress (Monmouth Junction, NJ, USA) and SM-102 from Biosynth Carbosynth (Staad, Switzerland). siRNA control (sense strand: 5′-AuGuAuuGGccuGuAuuAGdTsdT-3′; antisense strand: 5′-CuAAuAcAGGCcAAuAcAUdTsdT-3′) was provided by Axolabs GmbH (Kulmbach, Germany). Lipofectamine 3000, siSurvivin, and SYTOX™ Blue were purchased from Thermo Fisher Scientific (Schwerte, Germany).

### 3.2. Synthesis of LAF-XP Carriers

The carriers in this study were synthesized via SPPS, as detailed in Thalmayr et al. [36].

### 3.3. LNP Formulation

LNPs were prepared as described previously [40]. Briefly, an aqueous RNA-containing citrate buffer (10 mM, pH 4) was rapidly mixed with an ethanolic lipid phase at a volume ratio of 3:1 (*v*/*v*). After a 10-min incubation at room temperature (RT), the resulting LNP suspension was diluted with HBG buffer (20 mM HEPES, 5% (*w*/*v*) glucose, pH 7.4) to reduce the ethanol content to below 5%. The final RNA concentration in the LNP suspension was 5 μg/mL. The LNPs consisted of an ionizable component, DSPC, DMG-PEG2000, and cholesterol at molar ratios (%) of 22.2/15.6/2.3/59.9 (N/P 9) for XP-LNPs, 50/10/1.5/38.5 for SM102-LNP (N/P 6), and 50/10/1.5/38.5 for MC3-LNP (N/P 3). The N/P ratio was defined as the molar ratio of amine groups in the ionizable component to phosphate groups in the RNA. All components were dissolved in absolute ethanol prior to formulation.

Poly(I:C) and siSurvivin were used as functional cargos in all LNP formulations, while poly(I) and siCtrl were used to control for non-specific RNA and carrier-related effects.

### 3.4. Polyplex Formulation

The poly(I:C) and the LAF-XP carrier at an optimized N/P ratio were prepared separately by dilution in HBG buffer. To form the polyplexes, equal volumes of RNA and carrier solutions were mixed using rapid mixing, followed by incubation at RT for 40 min. In calculating the N/P ratio, all secondary amines from Stp units, as well as the terminal and tertiary amines of the LAFs, were considered. The applied N/P ratios for 1611, 1719, and 1752 were 18, 12, and 24, respectively. The final RNA concentration in the polyplex solution was 5 μg/mL.

### 3.5. Dynamic Light Scattering (DLS) and Electrophoretic Light Scattering (ELS) Measurements

Particle size, polydispersity index (PDI), and surface charge of the formulations were analyzed by DLS and ELS using a Zetasizer Nano ZS (Malvern Instruments, Malvern, Worcestershire, UK). Measurements of hydrodynamic diameter and PDI were performed in 80 μL of HBG buffer, with each sample analyzed in triplicate and each measurement consisting of six sub-runs. Before analysis, the samples were equilibrated at 25 °C for 30 s, with a refractive index of 1.330 and a viscosity of 0.8872 mPa·s. For electrophoretic mobility measurements, samples were diluted with HBG buffer to a total volume of 800 μL and analyzed three times, each measurement comprising 15 sub-runs following a 60 s equilibration period. Zeta potential values were calculated using the Smoluchowski equation.

### 3.6. RiboGreen Assay

The EE of RNA in LNPs was determined using the Quant-iT RiboGreen RNA Assay (Thermo Fisher Scientific, Schwerte, Germany), with fluorescence measurements performed on a Tecan microplate reader (Tecan, Männedorf, Switzerland). For sample preparation, 40 μL of each sample was diluted in 210 μL of RNase-free 1× TE buffer (10 mM Tris-HCl, 1 mM EDTA, pH 7.5). HBG buffer served as a blank. To distinguish encapsulated from total RNA, aliquots of the diluted samples (50 μL) were mixed with either 50 μL of 1× TE buffer (non-lysed LNPs) or 50 μL of 1× TE buffer supplemented with 2% (*v*/*v*) Triton X-100 (lysed LNPs). Samples were transferred in duplicate into a black 96-well plate and incubated at 37 °C for 10 min under constant shaking, then equilibrated at RT for 5 min. Subsequently, 100 μL of RiboGreen working solution (200-fold diluted in 1× TE buffer) was added to each well, and the plate was incubated at 37 °C for 15 min in the dark. Fluorescence intensity was then recorded at excitation and emission wavelengths of 485 nm and 520 nm, respectively. The EE was calculated by comparing fluorescence signals obtained from lysed and non-lysed LNPs and is reported as a percentage using the following formula:

$$EE\left(\%\right)=100-\frac{mean\;emission\;of\;untreated\;control}{mean\;emission\;of\;treated\;sample}\times 100\%$$

### 3.7. Agarose Gel Shift Assay

A 1% (*w*/*v*) agarose gel was prepared by dissolving agarose in hot 1× TBE buffer (10.8 g Tris base, 5.5 g boric acid, 0.75 g EDTA per liter of water, pH 8) under heating. After cooling to approximately 50 °C, Gel-Red dye was added, and the solution was cast into a gel tray with a comb. After complete solidification, the comb was removed. Prior to loading, each formulation was combined with loading buffer (6 mL glycerol, 1.2 mL 0.5 M EDTA, 2.8 mL H_2_O, and 0.02 g bromophenol blue) at a ratio of 5:1 (*v*/*v*) to obtain a final volume of 24 μL per sample. Following sample loading into the agarose gel, electrophoresis was conducted in 1× TBE running buffer at 120 V for 60 min. Free poly(I:C) in HBG buffer at a concentration equivalent to that used in the formulations was included as a positive control. After electrophoresis, nucleic acid bands were visualized by a UV transilluminator (Biostep, Jahnsdorf, Germany).

To examine the stability of LNPs, they were incubated in HBG with or without 45% (*v*/*v*) FBS for 2 h at 37 °C before electrophoresis.

### 3.8. TEM Measurement

Each LNP sample (10 µL) was applied for 30 s onto a carbon-coated copper grid (300 mesh, 3.0 mm O.D.; Ted Pella, Inc., Redding, CA, USA) that had been hydrophilized by argon plasma treatment at 420 V for 1 min. Excess solvent was removed using filter paper, and the grid was washed with 5 µL of 1% (*w*/*v*) uranyl formate. Negative staining was then performed immediately by applying an additional 5 µL droplet of uranyl formate for 5 s. The grid was air-dried at RT. Samples were imaged using a transmission electron microscope (JEOL, Tokyo, Japan) operated at 80 kV.

### 3.9. Cell Culture

The anti-tumoral effect of poly(I:C) was tested on human HeLa cervical adenocarcinoma, mouse N2A neuroblastoma, human LS174T colorectal carcinoma cells, human DU145 prostate cancer cells, and human HCT116 colorectal carcinoma cells. HeLa, N2A, and LS174T cells were cultured in DMEM (low glucose, 1 g/L), DU145 cells in RPMI-1640, and HCT116 cells in McCoy’s 5A medium. All media were supplemented with 10% (*v*/*v*) FBS, 100 U/mL of penicillin, and 100 µg/mL of streptomycin. All cells were maintained at 37 °C in a humidified atmosphere with 95% humidity/ 5% CO_2_.

PBMCs were isolated from the peripheral blood of healthy donors via density gradient centrifugation and cultured in RPMI-1640 supplemented with 10% FBS and IL-2 (160 IU/mL).

### 3.10. Cell Viability Measurement by MTT

Cells were seeded at a density of 4000 cells per well in 100 μL of culture medium and allowed to adhere overnight. The next day, cells were treated with formulations at the indicated RNA doses. HBG buffer served as a negative control. After incubation at 37 °C for the defined time point (48 or 72 h), 10 μL of MTT solution (5 mg/mL, Sigma-Aldrich) was added to each well, followed by a 2-h incubation. The supernatant was then carefully removed, and the plates were stored at −80 °C for at least 1 h. Formazan crystals formed in each well were dissolved in 100 μL of DMSO, then incubated at 37 °C for 30 min with gentle shaking to ensure complete solubilization. Absorbance was measured at 590 nm, with a reference wavelength of 690 nm, using a Tecan microplate reader. Cell viability was calculated as a percentage relative to HBG-treated cells (100%) and presented as the mean + standard deviation (SD) of triplicate wells.

### 3.11. Cell Viability by Flow Cytometry

PBMCs (pre-stimulated with 160 IU/mL IL-2 for 24 h) were seeded at a density of 4 × 10^5^ cells per well in 150 µL of culture medium and exposed to poly(I:C) (50 ng/well) using different formulations for 24 h. HBG served as a negative control. PBMC viability was assessed following treatment using a CytoFLEX flow cytometer (Beckman Coulter, Brea, CA, USA) with SYTOX™ Blue staining, and data were analyzed using FlowJo™ software (version 10.10, Ashland, OR, USA).

In a separate experiment, conditioned supernatants from PBMC cultures after poly(I:C) stimulation were collected and added to cancer cells (5 × 10^4^ LS174T cells per well). The supernatants were derived from PBMC cultures containing four times more cells than the recipient cancer cells, corresponding to a PBMC-to-cancer cell ratio of 4:1. After 24 h, cancer cell viability was measured.

### 3.12. Cell Apoptosis Measurement by Flow Cytometry

Cell apoptosis was assessed by flow cytometry using an apoptosis detection kit (Abcam, Cambridge, UK) according to the manufacturer’s instructions. Briefly, cancer cells were seeded at a density of 12,000 cells per well and, the following day, treated with LNPs encapsulating either siSurvivin (5 ng per 4000 cells) or poly(I:C) (1 ng per 4000 cells). After a 72-h incubation, cells were harvested, resuspended in binding buffer containing Annexin V-FITC and PI, and incubated for 5 min at RT in the dark prior to flow cytometry analysis.

### 3.13. Quantitative Reverse Transcription Polymerase Chain Reaction (qRT-PCR)

Cells were plated at a density of 1 × 10^6^ cells. The next day, they were treated with siSurvivin-LNPs at the indicated doses. After a 48-h incubation, cells were trypsinized, total mRNA was extracted using the peqGOLD total RNA Kit (VWR, Darmstadt, Germany), and cDNA was synthesized using the qScript cDNA Synthesis Kit (Quantabio, Beverly, MA, USA), both according to the manufacturers’ protocols. qRT-PCR was performed on a LightCycler^®^ 480 system (Roche, Basel, Switzerland) using TaqMan probes and the Gene Expression Master Mix (Cat. No. 4331182, Thermo Fisher Scientific, Waltham, MA, USA), with Glyceraldehyde-3-phosphate dehydrogenase (GAPDH) as a housekeeping gene. Each sample contained 5 μL of Master Mix, 0.5 μL of probe, and 4.5 μL of diluted cDNA (1:10 diluted in RNase-free water). PCR reactions were carried out for 10 min at 95 °C, followed by 45 cycles of 95 °C for 15 s, 60 °C for 15 s, and 72 °C for 15 s. Results were analyzed using the 2^−ΔΔCt^ method for relative gene quantification.

### 3.14. CXCL10 Quantification by ELISA

Cancer cells were seeded at a density of 12,000 cells per well and incubated overnight. Cells were then exposed to poly(I:C)-loaded formulations (10 and 5 ng per 4000 cells) for 24 h, after which culture supernatants were collected, cleared by centrifugation to remove cell debris, and subjected to CXCL10 quantification using a Human CXCL10/IP-10 Quantikine ELISA Kit (Cat. No. DIP100; R&D Systems, Wiesbaden-Nordenstadt, Germany) according to the manufacturer’s instructions. HBG was used as a negative control. Absorbance was measured at 450 nm with wavelength correction at 540 or 570 nm using a Tecan microplate reader, and concentrations were calculated from standard curves.

### 3.15. IFN-γ and GrB Quantification by ELISA

Following IL-2 pre-stimulation (160 IU/mL, 24 h), PBMCs were seeded at a density of 4 × 10^5^ cells per well and stimulated with poly(I:C) (50 ng/well) using different formulations for 24 h. Cell-free supernatants were obtained by centrifugation (2000× *g*, 10 min) and analyzed for GrB and IFN-γ using Human Quantikine ELISA Kits (Cat. No. DGZB00 and DIF50C, respectively; R&D Systems, Wiesbaden-Nordenstadt, Germany) according to the manufacturers’ instructions. HBG was used as a negative control. Absorbance was recorded at 450 nm with wavelength correction at 540 or 570 nm using a Tecan microplate reader.

### 3.16. Analysis of Cell Surface Marker Expression by Flow Cytometry

PBMCs were pre-stimulated with IL-2 (160 IU/mL) for 24 h prior to seeding at a density of 4 × 10^5^ cells per well. Cells were subsequently exposed to poly(I:C) (50 ng per well) using various formulations for 24 h, with HBG buffer-treated cells serving as a negative control. After treatment, cells were harvested and stained with monoclonal antibodies (mAb), including PE-conjugated anti-CD56 (Clone N901; Beckman Coulter, Brea, CA, USA), PE-Cy7-conjugated anti-CD3 (Clone HIT3a; BioLegend, San Diego, CA, USA), FITC-conjugated anti-LAMP-1/CD107a (Clone H4A3; Miltenyi Biotec, Bergisch Gladbach, Germany), and APC-conjugated anti-CD69 (Clone FN50; BioLegend, San Diego, CA, USA). Surface marker expression (CD69 and LAMP-1) was quantified in viable, SYTOX™ Blue-negative cells using a flow cytometer, and data were analyzed with FlowJo™ software (version 10.10).

### 3.17. Statistical Analysis

Statistical analyses were performed using the unpaired Student’s *t*-test, one-way analysis of variance (ANOVA) in GraphPad Prism (Version 9.5.1, GraphPad, San Diego, CA, USA). *p* values were considered statistically significant as follows: ns: not significant, * *p* ≤ 0.05, ** *p* ≤ 0.01, *** *p* ≤ 0.001, and **** *p* ≤ 0.0001.

## 4. Conclusions

Successful therapy is determined not only by the choice of therapeutic cargo but also by how it is delivered to the site of action. While poly(I:C) has already shown considerable promise in cancer treatment, unlocking its full potential prompted us to develop poly(I:C)-LNP formulations based on recently developed, efficient bundle LAF_4_-Stp_1_ carriers. These lipopeptide-like structures integrate four units of lipophilic cationizable LAF domains and one unit of hydrophilic cationizable Stp. Their excellent performance in vitro and in vivo is attributed to their double pH-responsive behavior, which leads to high cellular uptake, robust endosomal escape, and efficient functional delivery. In this work, as a result of effective transfection by bundle XP-LNPs, poly(I:C) imposed pronounced cell death in a range of human cancer cell lines, including glioblastoma, prostate, cervical, and colorectal cancers, often at lower doses and with greater potency than Lipofectamine and LNPs formulated with FDA-approved ionizable lipids SM102 and MC3. The potential of bundle XP was also evident upon poly(I:C) delivery into PBMCs, with stimulation occurring without cytotoxicity, not only at the level of surface activation markers but also in cytokine production. Supernatants from poly(I:C)-stimulated PBMC cultures reduced tumor cell viability, likely due to a cocktail of PBMC-released effector molecules and possibly leftover LNPs. Induction of direct tumor cell killing and immune activation requires a delivery platform capable of efficiently targeting different cell types, as demonstrated in our study. Another noteworthy finding is the combinatorial effect of poly(I:C) with survivin knockdown, which together produce a notable anti-tumor response at very low doses, either sequentially, where siRNA-mediated knockdown induces sensitization, or concurrently, where additive or potentially synergistic effects are observed. Collectively, the bundle XP-LNP (1755-LNP) provides a flexible platform to support a multi-layered strategy, including direct tumor killing by intracellular poly(I:C) delivery into cancer cells, tumor sensitization through survivin silencing in combination with poly(I:C), and immune-mediated effects via activation of PBMCs, leading to IFN-γ and GrB production.

## Data Availability

Data are contained within this article and the Supplementary Materials.

## Supplementary Materials

The following supporting information can be downloaded at: https://www.mdpi.com/article/10.3390/ijms27114968/s1.

## Abbreviations

The following abbreviations are used in this manuscript:

ANOVA	Analysis of variance
CXCL10	C-X-C motif chemokine ligand 10
DCs	Dendritic cells
DLS	Dynamic light scattering
DMEM	Dulbecco’s Modified Eagle Medium
DMG-PEG2000	Dimyristoyl glycerol polyethylene glycol 2000
DSPC	1,2-distearoyl-sn-glycero-3-phosphocholine
EDTA	Disodium ethylenediaminetetraacetic acid
EE	Encapsulation efficiency
ELISA	Enzyme-linked immunosorbent assay
ELS	Electrophoretic light scattering
FBS	Fetal bovine serum
GrB	Granzyme B
HBG	HEPES-buffered glucose
IFN-γ	Interferon gamma
LAF	Lipoamino fatty acid
LNP	Lipid nanoparticle
MC3	DLin-MC3-DMA
MDA5	Melanoma differentiation-associated gene 5
MTT	3-(4,5-dimethylthiazol-2-yl)-2,5-diphenyltetrazolium bromide assay
N/P ratio	Nitrogen-to-phosphate ratio
NK cell	Natural killer cell
PBMCs	Peripheral blood mononuclear cells
PDI	Polydispersity index
Poly(I)	Polyinosinic acid
Poly(I:C)	Polyinosinic:polycytidylic acid
qRT-PCR	Quantitative reverse transcription polymerase chain reaction
RIG-I	Retinoic acid-inducible gene I
RLRs	RIG-I-like receptors
RNAi	RNA interference
RT	Room temperature
siCtrl	Control small interfering RNA
siRNA	Small interfering RNA
siSurvivin	Survivin-targeting small interfering RNA
SPPS	Solid-phase peptide synthesis
Stp	Succinoyl tetraethylene pentamine
TEM	Transmission electron microscopy
TLR3	Toll-like receptor 3
XP	Xenopeptide
