# Supplementary material for: Poly(I:C) Lipoamino Bundle LNPs Induce Tumor Cytotoxicity and Immune Activation with Enhanced Efficacy by Survivin Silencing
Source: Int J Mol Sci. 2026 May 30;27(11):4968. doi: 10.3390/ijms27114968 (PMC13256650; doi:10.3390/ijms27114968)
Supplement: Supplementary file 1 [file ijms-27-04968-s001.zip › ijms-4311918-supplementary.pdf]

Poly(I:C) Lipoamino Bundle LNPs Induce Tumor Cytotoxicity and Immune Activation with Enhanced Efficacy by Survivin Silencing

Mina Yazdi[1,2,*], Zahra Hasheminejad[1†], Khouloud Hachani[3†], Joyce Kache[3], Melina Grau[1], Barbara Wollenberg[3], Ali Bashiri Dezfouli[3], and Ernst Wagner[1,2,*]

[1] Pharmaceutical Biotechnology, Department of Pharmacy, Ludwig-Maximilians-Universität (LMU), 81377 Munich, Germany; zahrahasheminezhad.khu@gmail.com (Z.H.); melina.grau@cup.uni-muenchen.de (M.G.)

[2] CNATM—Cluster for Nucleic Acid Therapeutics Munich, 81377 Munich, Germany

[3] Department of Otolaryngology, Head and Neck Surgery, TUM School of Medicine and Health, Technical University of Munich, 81675 Munich, Germany; khouloud.hachani@tum.de (K.H.); kachejoyce139@gmail.com (J.K.); barbara.wollenberg@tum.de (B.W.); ali.bashiri@tum.de (A.B.D.)

***** Correspondence: mina.yazdi@cup.uni-muenchen.de (M.Y.); ernst.wagner@lmu.de (E.W.)

† These authors contributed equally to this work.

Supplementary Materials

Figures 2

Figures

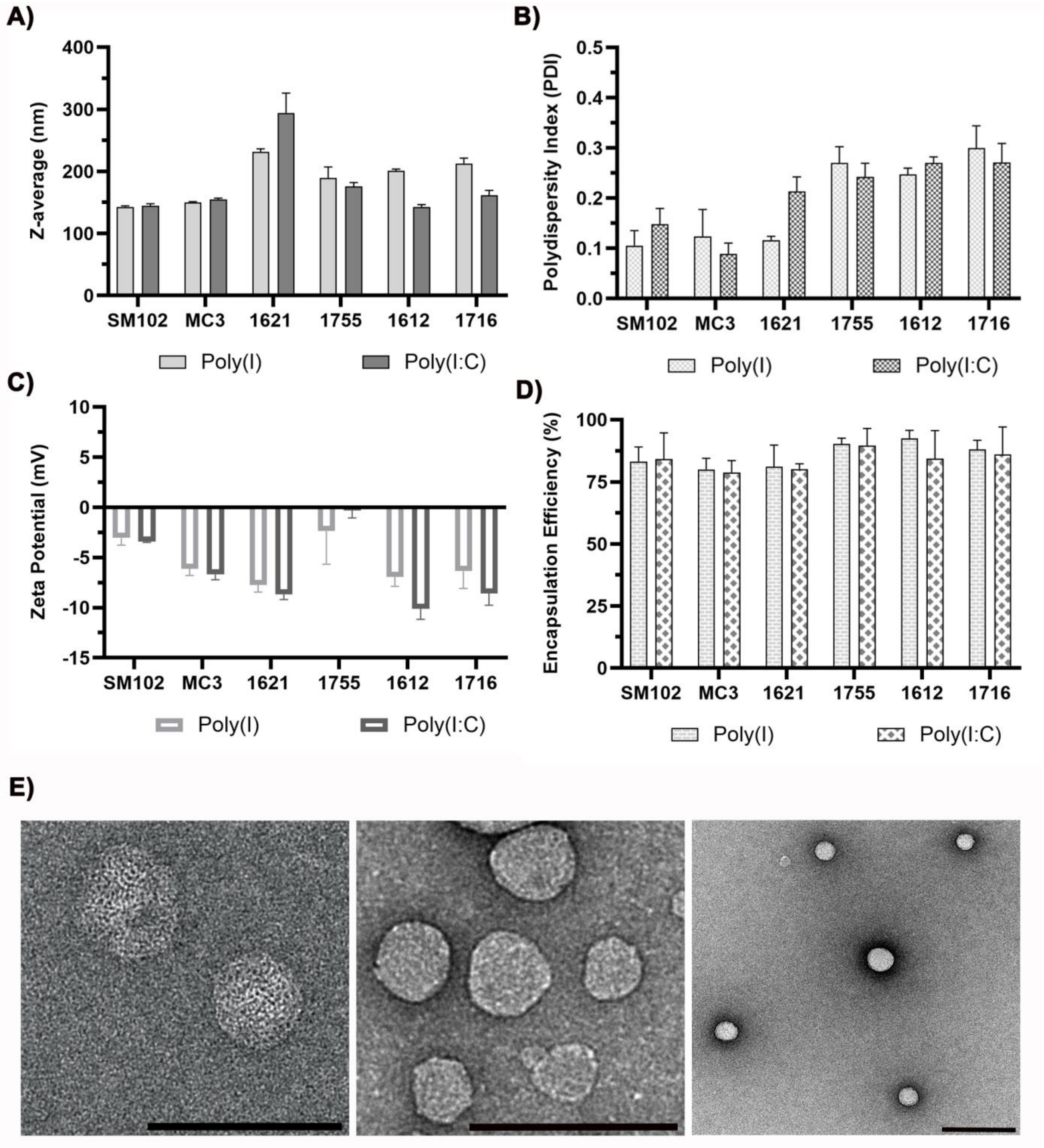

Figure S1. Physicochemical characterization of poly(I:C)-loaded XP-LNPs. LNPs were formulated as described in the *Experimental Section*. The standard SM102- and MC3-LNPs served as reference controls. In parallel, poly(I)-LNPs were included to assess the impact of RNA structure on nanoparticle properties. **A)** The z-average diameter (nm), **B)** polydispersity index (PDI), and **C)** zeta potential (mV) of LNPs evaluated by dynamic and electrophoretic light scattering (DLS and ELS). **D)** RNA encapsulation efficiency (EE) in the LNPs measured using RiboGreen assay. The data are expressed as mean + SD (n = 3). **E)** Representative TEM images of poly(I:C)-LNPs formulated, from left to right, with the bundles 1621 (8Oc) and 1755 (14He), and the U-shape 1612 (12Oc). The scale bar is 200 nm.

A)

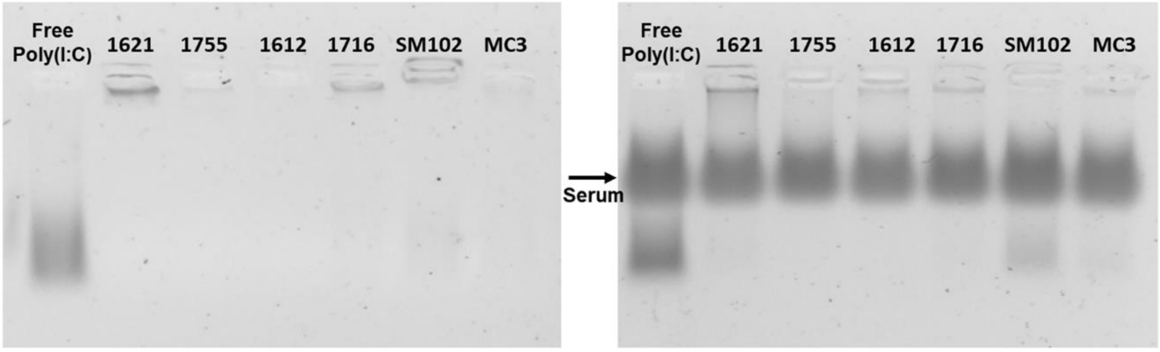

B)

	Z-average (nm)		PDI	
**LNP ID**	**Day 1**	**Day 6**	**Day 1**	**Day 6**
**1621**	252.97 ± 3.05	279.07 ± 3.33	0.19 ± 0.022	0.21 ± 0.006
**1755**	160.87 ± 3.13	157.10 ± 3.68	0.19 ± 0.015	0.25 ± 0.036
**1612**	123.60 ± 3.76	125.10 ± 4.75	0.30 ± 0.036	0.26 ± 0.043
**1716**	120.40 ± 2.60	146.73 ± 1.86	0.22 ± 0.002	0.20 ± 0.057
**SM102**	138.43 ± 2.87	162.43 ± 1.3	0.10 ± 0.014	0.18 ± 0.047
**MC3**	105.47 ± 1.31	138.70 ± 1.62	0.14 ± 0.005	0.19 ± 0.010

Figure S2. Stability of poly(I:C)-loaded XP-LNPs. LNPs were formulated as described in the *Experimental Section* and were subjected to different stability measurements. **A)** Stability of LNPs determined by agarose gel electrophoresis. Before the gel run, LNPs were incubated in the preparation buffer in the absence or presence of fetal bovine serum (FBS; 45% v/v) for 2 h at 37 °C. Free poly(I:C) at a concentration similar to that used in the LNPs was used as a positive control. **B)** The particle size and PDI of LNPs measured by DLS after incubation for 6 days at 4 °C. The data are expressed as mean + SD (n = 3).

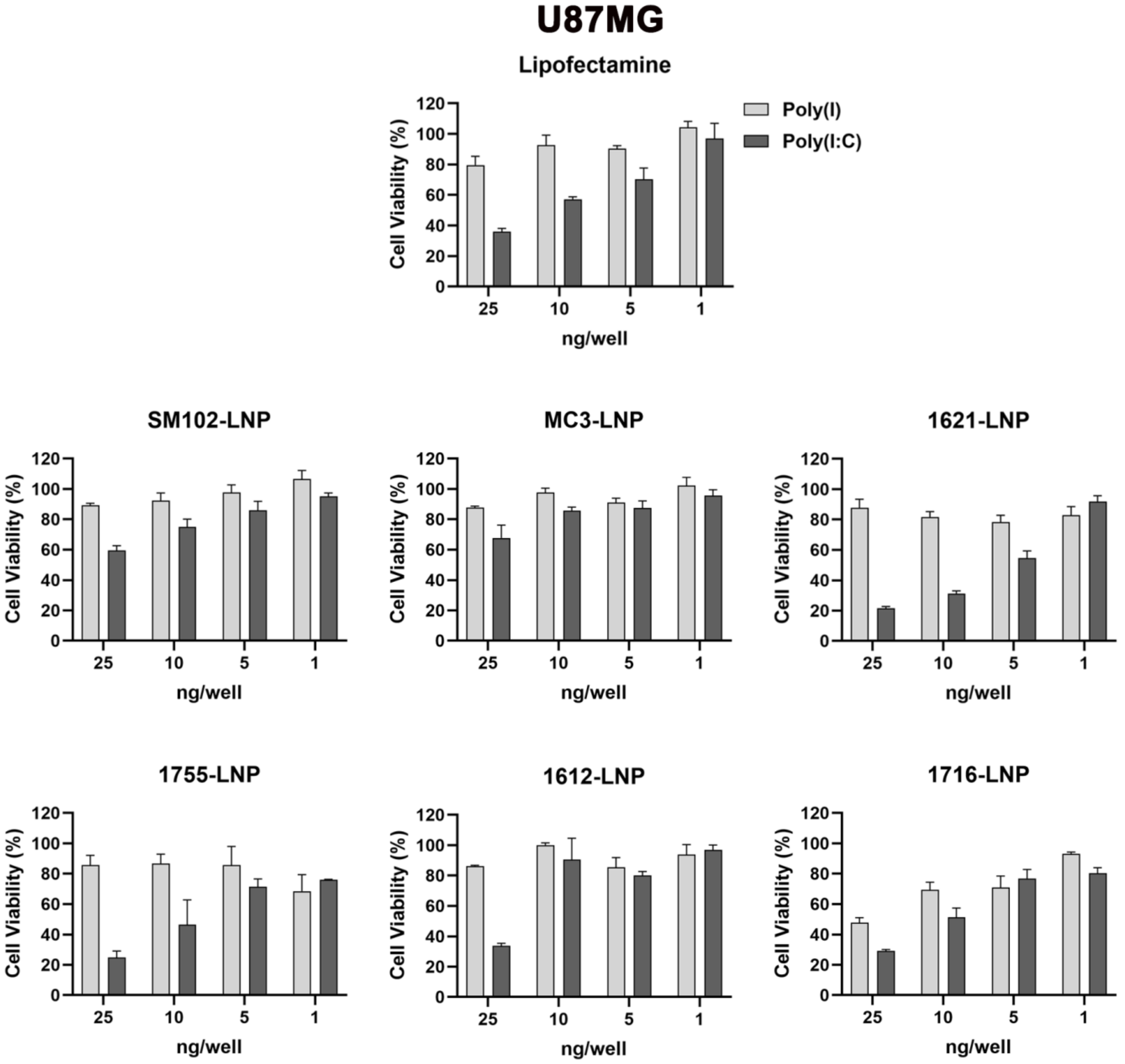

Figure S3. **The effect of poly(I:C)-encapsulated XP-LNPs on human U87MG glioblastoma cell line**. LNPs were formulated as described in the *Experimental Section*. Lipofectamine, SM102-, and MC3-LNPs were included as comparative delivery controls. In parallel, poly(I)-loaded formulations were used to account for non-specific effects. U87MG cells (4000 cells per well containing 100 µL culture medium) were treated with different doses of poly(I:C) (25, 10, 5, and 1 ng per well) for 48 h. Cell viability was assessed using MTT assay and is expressed as a percentage relative to buffer-treated cells (mean + SD, *n* = 3). Poly(I:C)-mediated cytotoxicity, obtained from the difference between poly(I) and poly(I:C) effects on cell viability, is presented in the main **Figure 1A**.

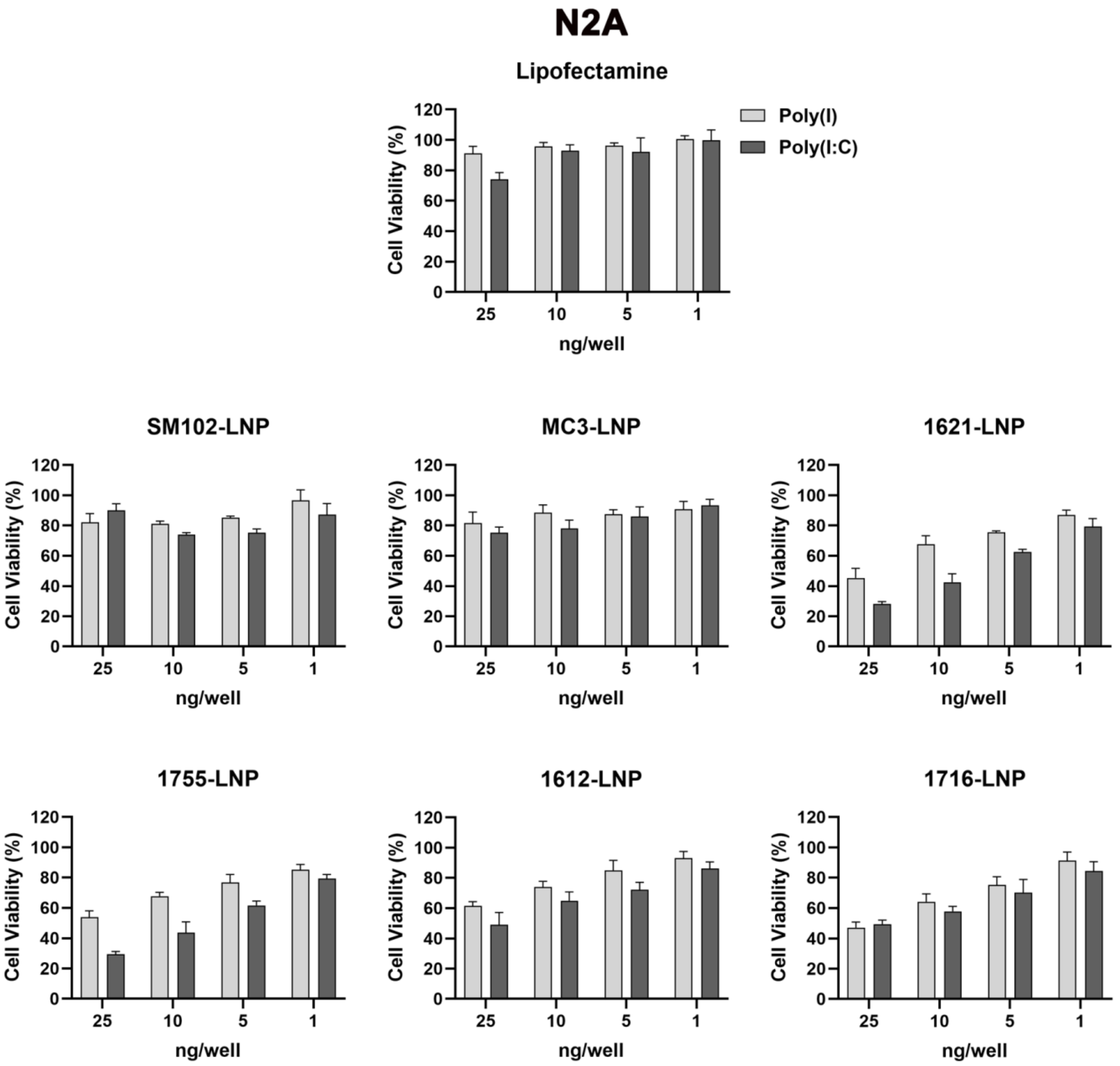

Figure S4. The effect of poly(I:C)-encapsulated XP-LNPs on mouse N2A neuroblastoma cell line. The experiment and data analysis were performed analogously as described for **Figure S3.**

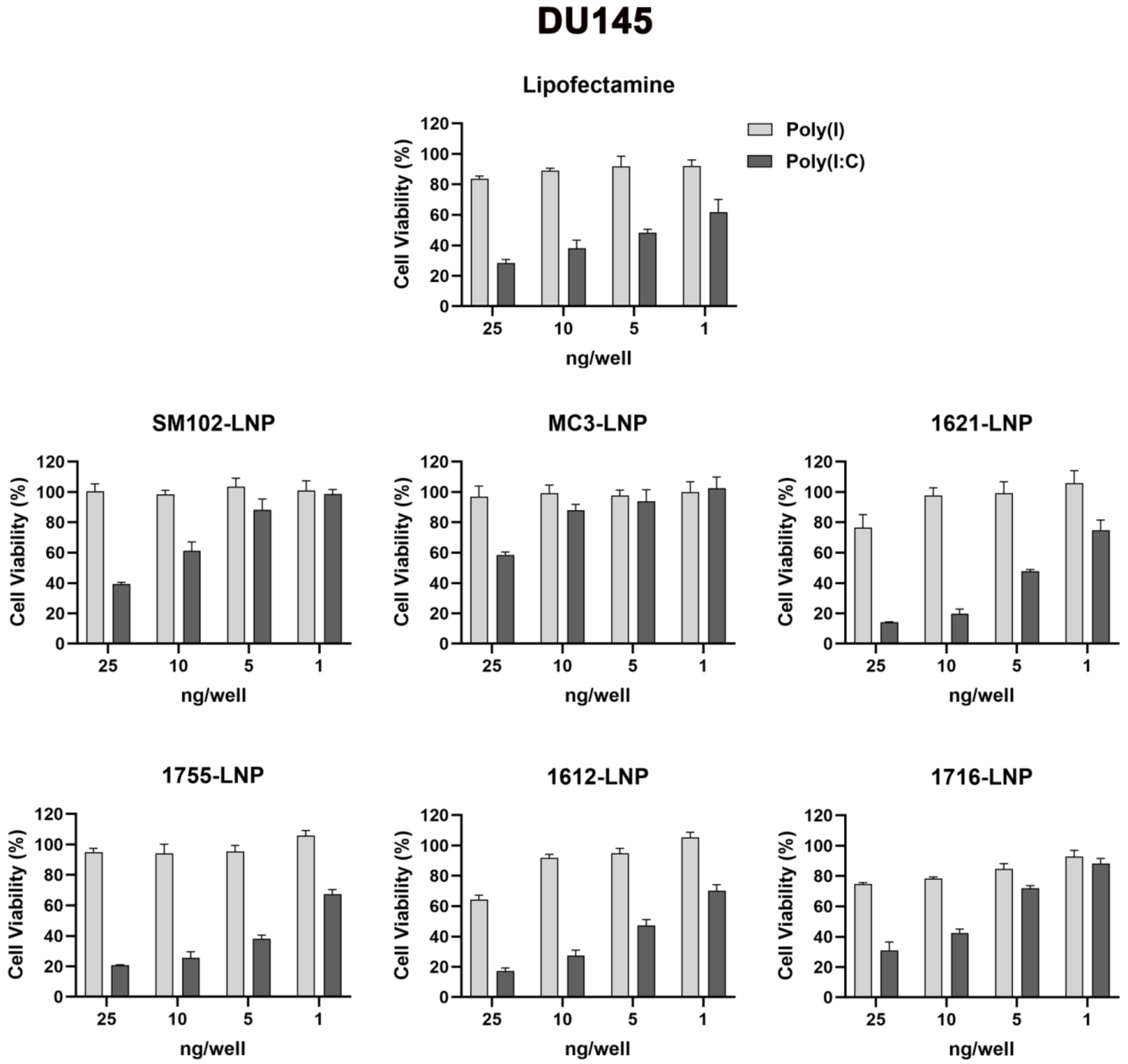

Figure S5. The effect of poly(I:C)-encapsulated XP-LNPs on human DU145 prostate cancer cell line. The experiment and data analysis were performed analogously as described for **Figure S3.** Poly(I:C)-mediated cytotoxicity is presented in the main **Figure 1B**.

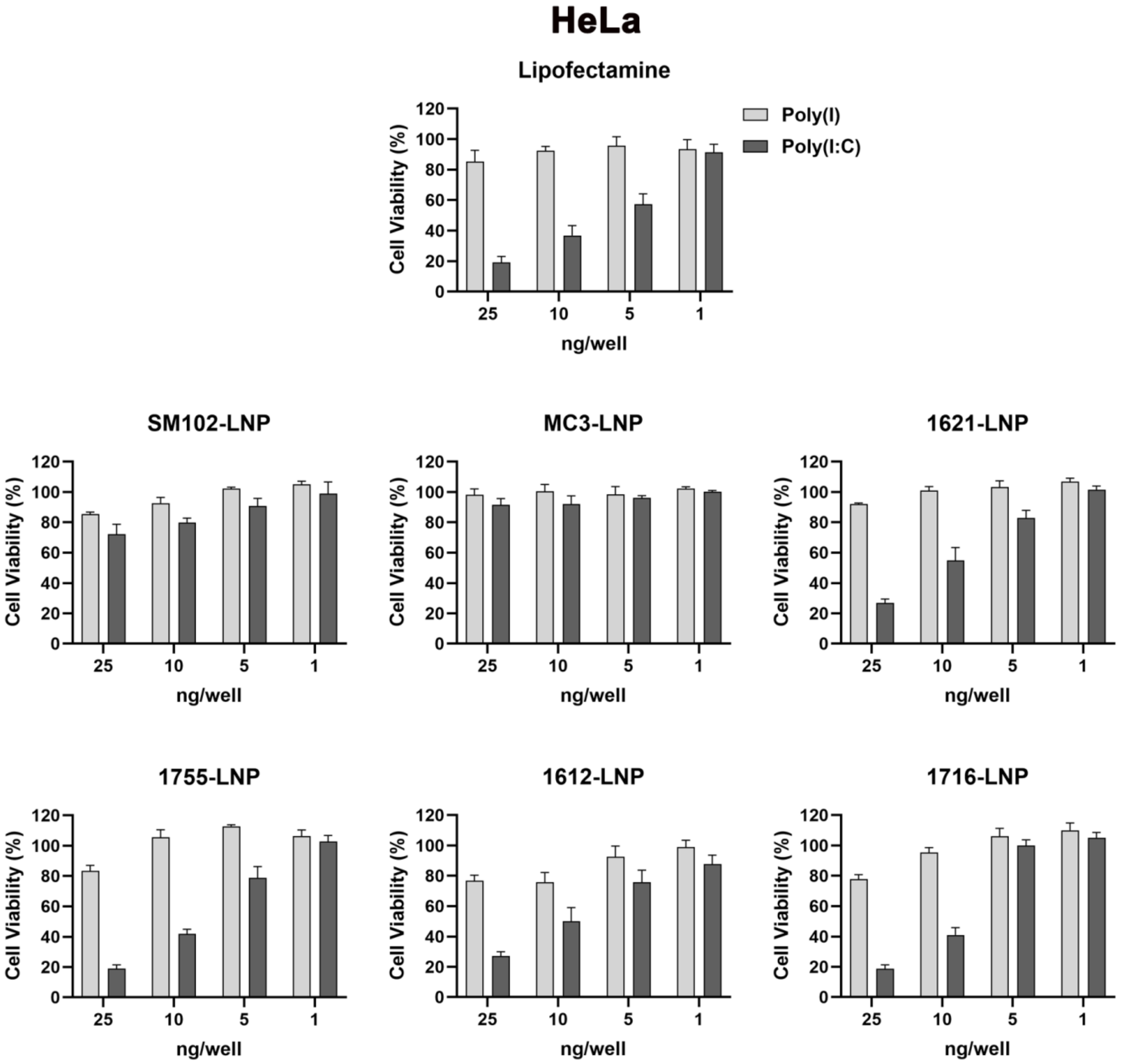

Figure S6. The effect of poly(I:C)-encapsulated XP-LNPs on human HeLa cervical adenocarcinoma cell line. The experiment and data analysis were performed analogously as described for **Figure S3.** Poly(I:C)-mediated cytotoxicity is presented in the main **Figure 1C**.

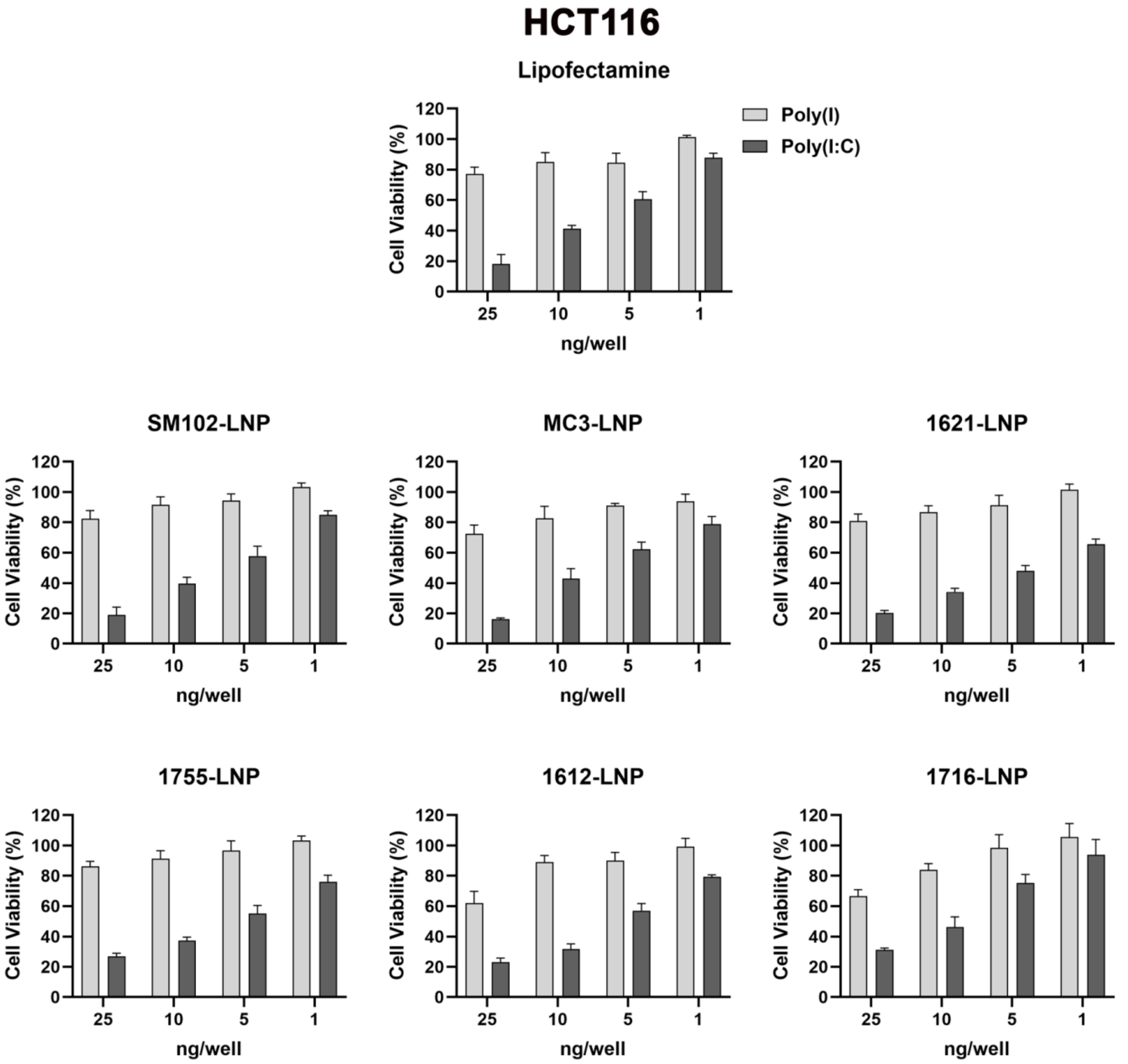

Figure S7. The effect of poly(I:C)-encapsulated XP-LNPs on human HCT116 colorectal carcinoma cell line. The experiment and data analysis were performed analogously as described for **Figure S3.** Poly(I:C)-mediated cytotoxicity is presented in the main **Figure 1D**.

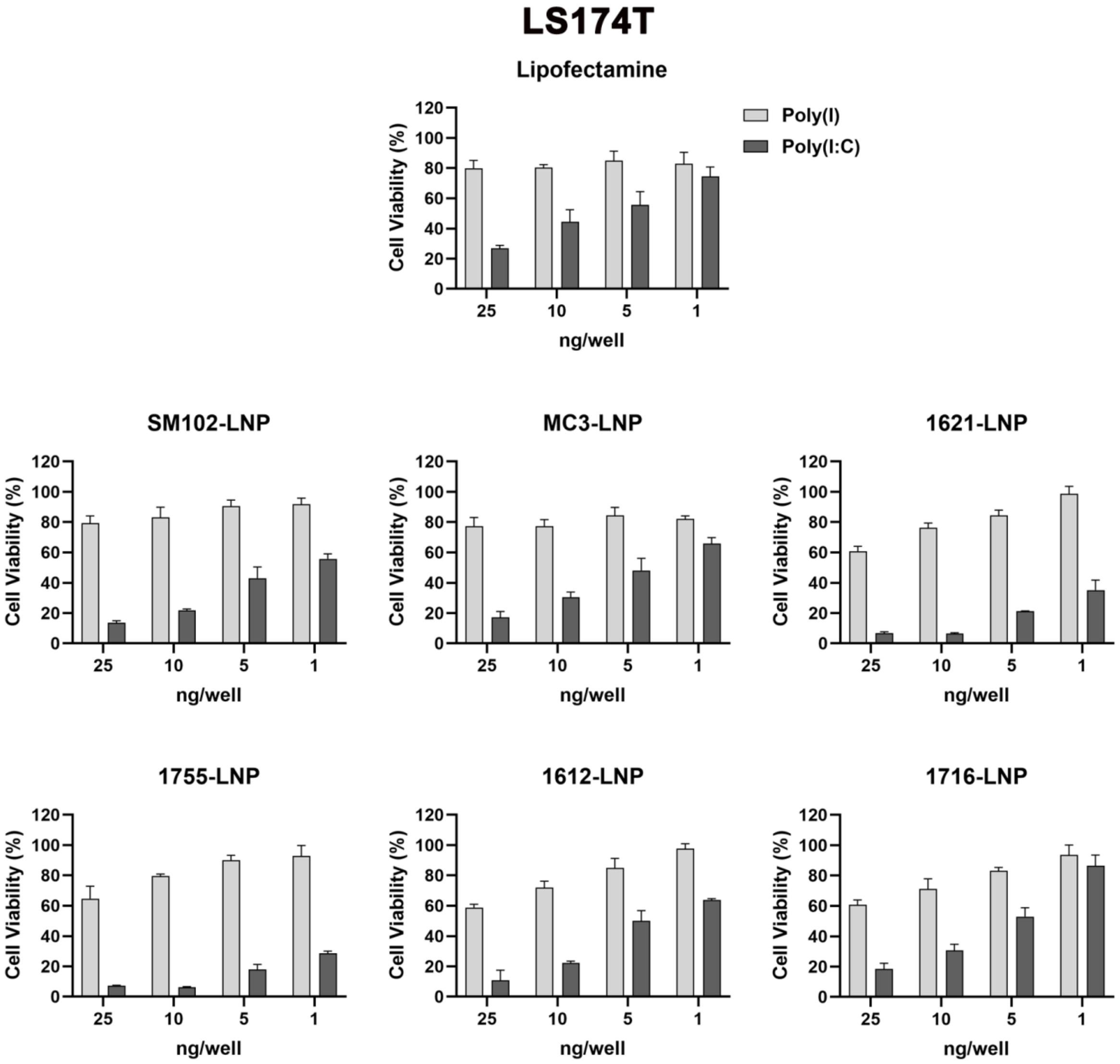

Figure S8. The effect of poly(I:C)-encapsulated XP-LNPs on human LS174T colorectal carcinoma cell line. The experiment and data analysis were performed analogously as described for **Figure S3.** Poly(I:C)-mediated cytotoxicity is presented in the main **Figure 1E**.

A)

Topologies	ID	Sequence (N→C)
**Bundle**		
	1752	K[K(12Bu)$_2$]$_2$-Stp
**U-shape**		
U1	1611	K(12Oc)-Stp-K(12Oc)
U1	1719	[K(12Oc)]$_2$-Stp$_2$-[K(12Oc)]$_2$

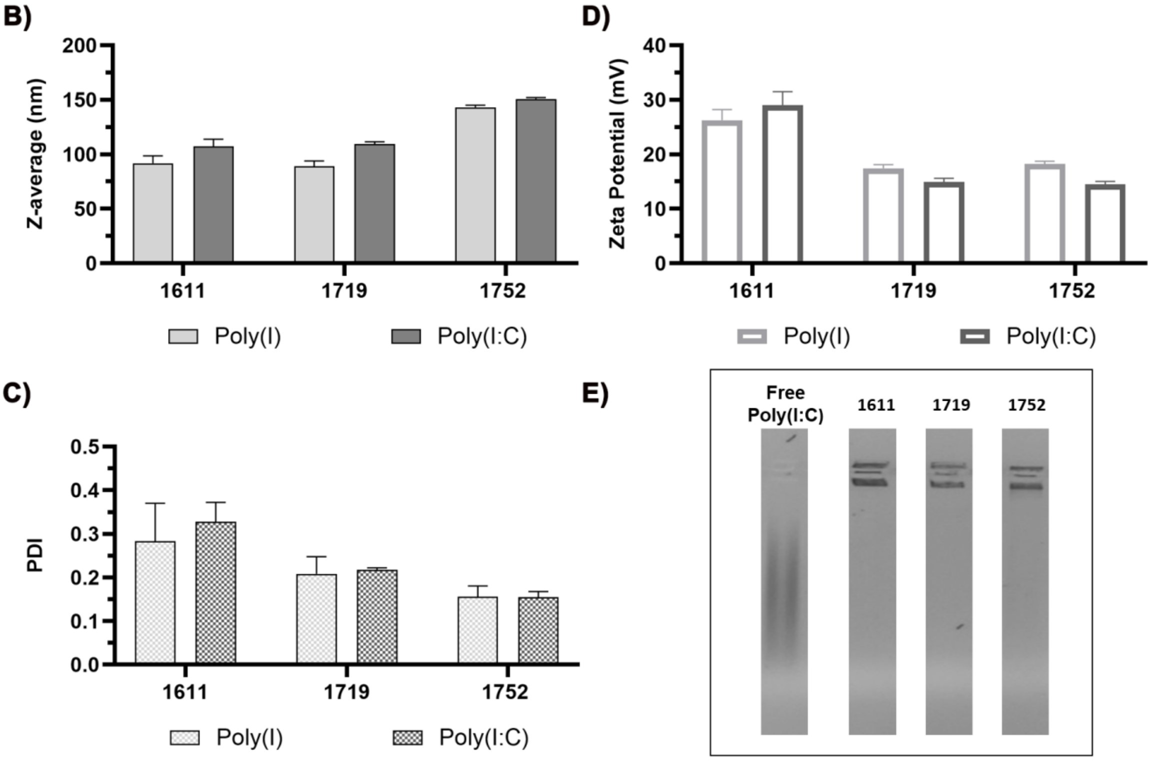

Figure S9. Physicochemical characterization of poly(I:C)-polyplexes. Polyplexes (PPs) were formed with LAF-XPs as described in the *Experimental Section*. **A)** Selected LAF-XP candidates for complexation with either poly(I) or poly(I:C) at their optimal N/P ratios (1611 at N/P 18, 1719 at N/P 12, and 1752 at N/P 24). **B)** The z-average diameter (nm), **C)** PDI, and **D)** zeta potential (mV) of PPs evaluated by DLS and ELS. The data are expressed as mean + SD (*n* = 3). **E)** Poly(I:C) binding stability within polyplexes determined by agarose gel electrophoresis. Free poly(I:C) was used as a positive control.

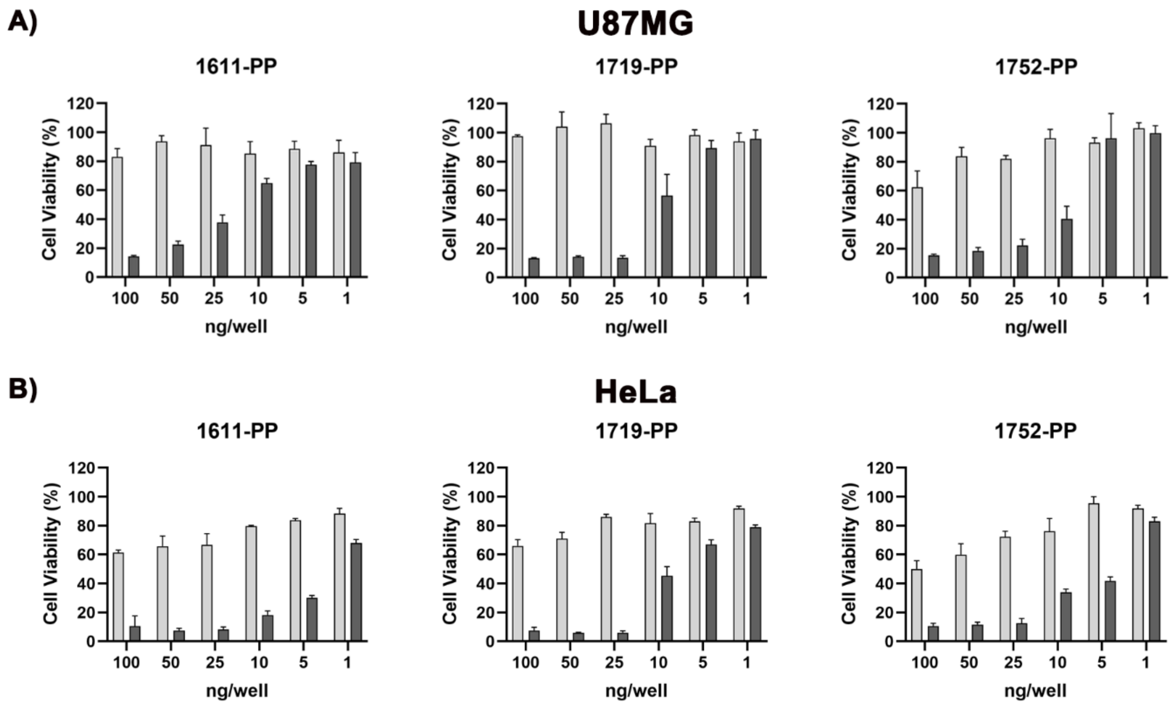

Figure S10. **The effect of poly(I:C)-polyplexes on human cancer cells.** PPs were formed with LAF-XPs as described in the *Experimental Section*. Poly(I)-loaded PPs were used to account for non-specific effects. **A)** U87MG and **B)** HeLa cells (4000 cells per well containing 100 µL culture medium) were treated with different doses of poly(I:C) (100, 50, 25, 10, 5, and 1 ng per well) for 48 h. Cell viability was assessed using MTT assay and is expressed as a percentage relative to buffer-treated cells (mean + SD, *n* = 3).

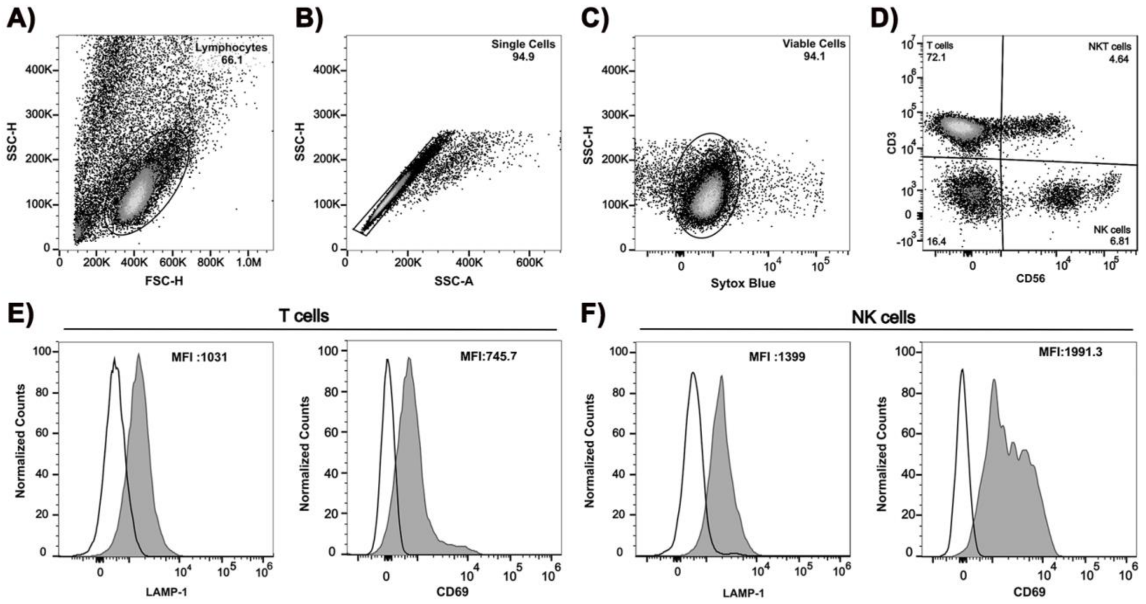

Figure S11. Representative flow cytometric gating strategy for the analysis of CD69 and LAMP-1 expression on T and NK cells in peripheral blood mononuclear cells (PBMCs). Cells were gated based on **A)** forward and side scatter, **B)** singlet selection, and **C)** viable cells identified as SYTOX™ Blue-negative. **D)** T cells and NK cells were then discriminated based on CD3 and CD56 expression. The expression of CD69 and LAMP-1 was assessed in **E)** T cell and **F)** NK cell populations using fluorescent-labeled monoclonal antibodies (mAb) (gray histograms). Isotype-matched controls (white histograms) were used as negative controls. Data are presented as mean fluorescence intensity (MFI).

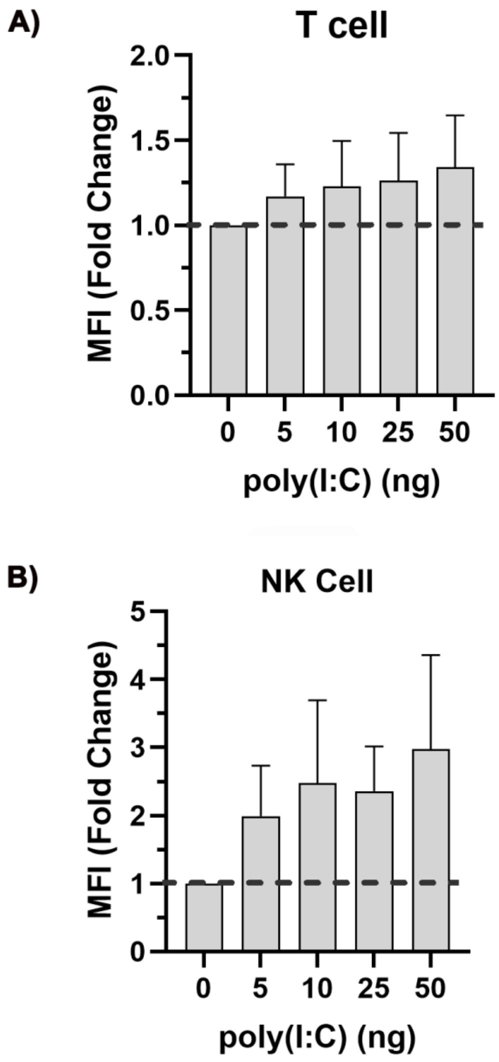

Figure S12. Effect of XP-LNP-encapsulated poly(I:C) on CD69 expression in PBMCs. 1755-LNP was formulated as described in the *Experimental Section*. Healthy-donor isolated PBMCs (4×10^5 cells) were treated with different doses of poly(I:C) (50, 25, 10, and 5 ng) for 24 h prior to cell surface analysis in **A)** T cells and **B)** NK cells by flow cytometry. MFI of CD69 in each treated sample is reported as a fold change of the buffer-treated sample (mean + SD from three independent donors, each in duplicate).

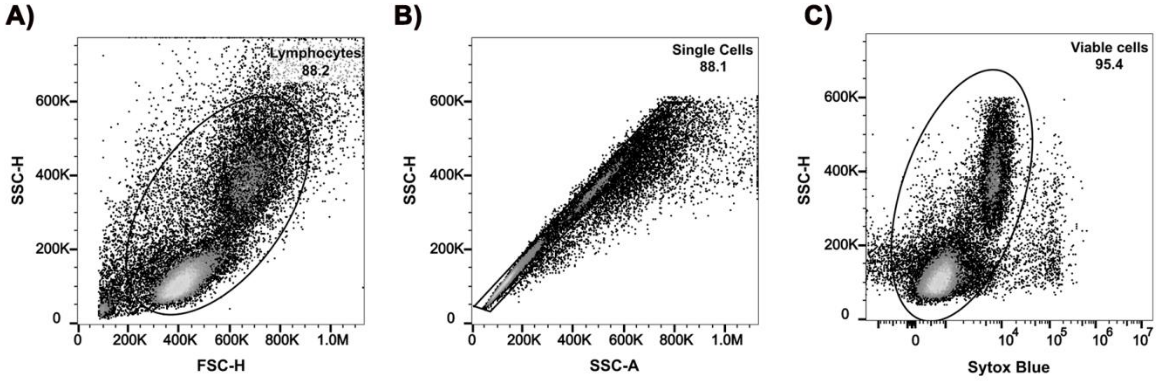

Figure S13. Representative flow cytometric gating strategy for viability assessment of PBMCs. Cells were gated based on **A)** forward and side scatter, **B)** singlet selection, and **C)** viable cells were defined as SYTOX™ Blue-negative cells. Data are presented as the percentage of SYTOX™ Blue-negative cells.

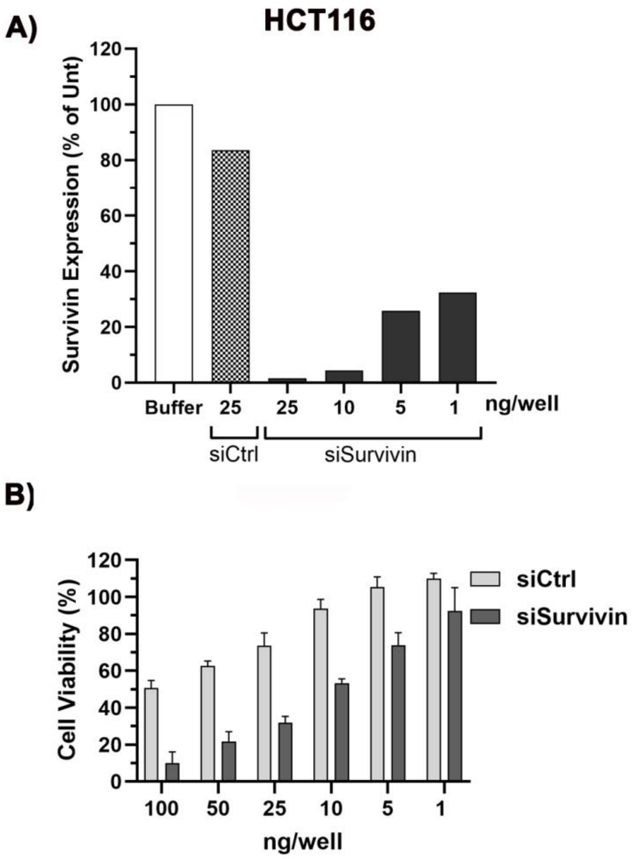

Figure S14. The effect of survivin knockdown on HCT116 cells via XP-LNP-mediated delivery. 1755-LNP encapsulating siSurvivin was formulated as described in the *Experimental Section*. siCtrl-loaded LNP was used to account for non-specific effects. **A)** siRNA-mediated gene silencing of survivin quantified by qRT-PCR. Cells were treated with different doses of siSurvivin (25, 10, 5, and 1 ng per 4000 cells) for 48 h. siCtrl was tested at a dose of 25 ng. Total mRNA of each treatment group was extracted and used for cDNA synthesis, followed by qRT-PCR analysis. Gene expression levels were quantified using the $2^{-\Delta\Delta Ct}$ method relative to buffer-treated cells, defined as untreated (Unt) cells in the graph, with GAPDH as the housekeeping gene. **B)** Viability of siRNA-treated HCT116 cells assessed by MTT assay. The cells (4000 cells per well containing 100 µL culture medium) were treated with different doses of siRNA (100, 50, 25, 10, 5, and 1 ng per well) for 72 h. The data are expressed as a percentage relative to buffer-treated cells (mean + SD, *n* = 3).

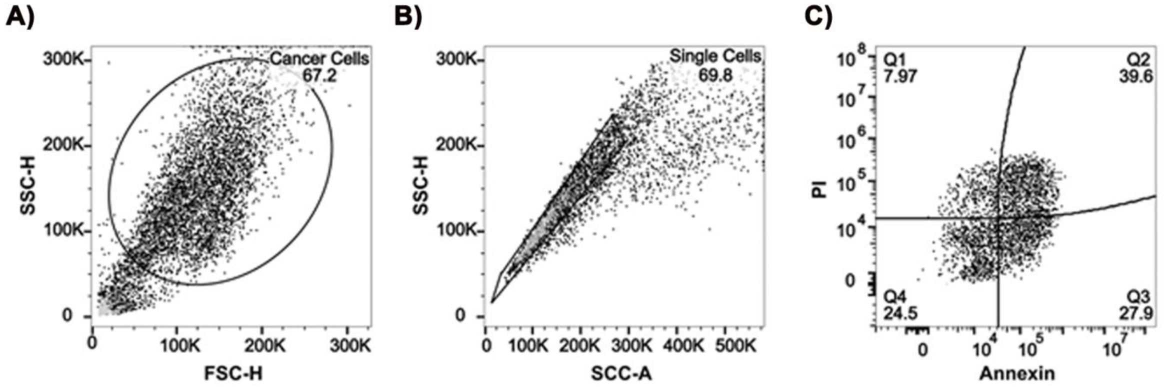

Figure S15. Representative flow cytometric gating strategy for apoptosis assessment in HCT116 cells after poly(I:C) treatment. Cancer cell population was gated based on **A)** forward and side scatter, **B)** singlet selection, and **C)** Annexin V-FITC (FL-1 channel) vs PI (FL-3 channel) staining. Cell populations were defined as follows: viable cells (Annexin V^{-}/PI^{-}), early apoptotic cells (Annexin V^{+}/PI^{-}), late apoptotic cells (Annexin V^{+}/PI^{+}), and necrotic cells (Annexin V^{-}/PI^{+}). The percentage of each population was determined within the final gated single-cell population.

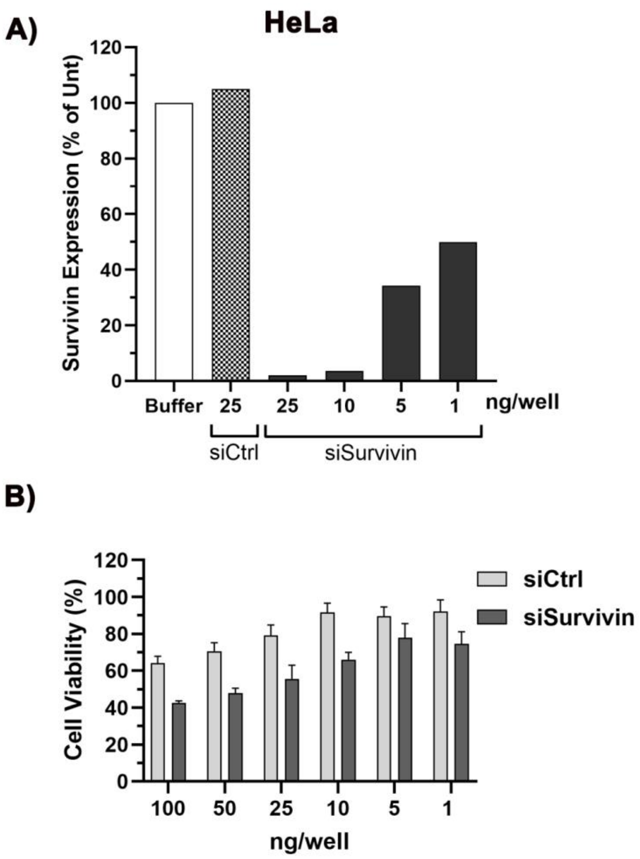

Figure S16. The effect of survivin knockdown on HeLa cells using XP-LNP. The experiment and data analysis were performed analogously as described for **Figure S14. A)** siRNA-mediated gene silencing of survivin measured by qRT-PCR. **B)** Viability of siRNA-treated HeLa cells assessed by MTT assay.

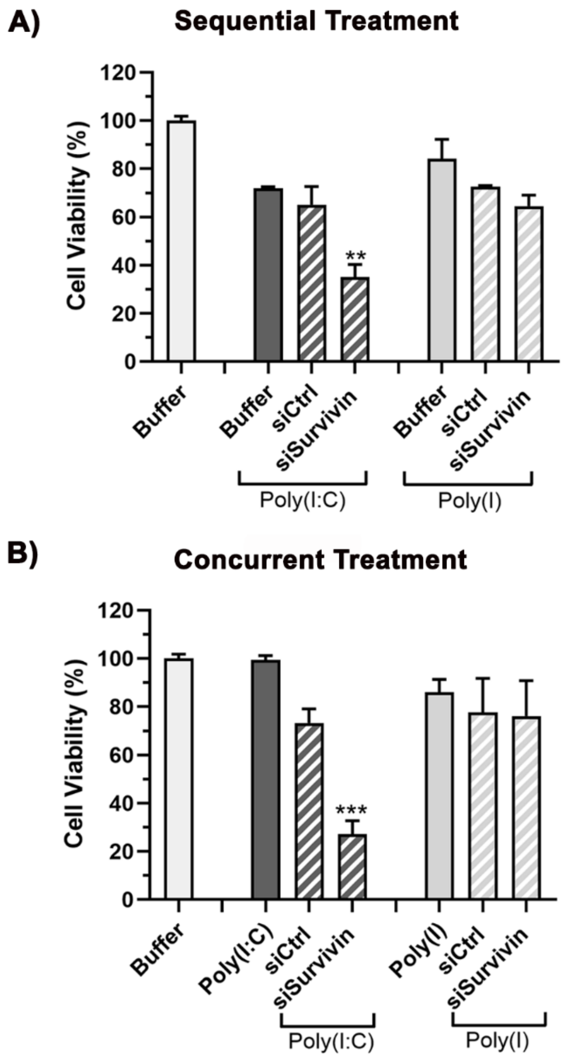

Figure S17. Effect of XP-LNP-mediated delivery of survivin siRNA and poly(I:C) on the viability of HeLa cells. 1755-LNP was formulated as described in the *Experimental Section*. For sequential treatment, HeLa cells were first treated with 1755-LNP encapsulating siSurvivin (5 ng per 4000 cells) for 24 h, followed by poly(I:C)-LNP (1 ng per 4000 cells) for an additional 48 h. For concurrent treatment, cells were co-treated with siSurvivin-LNP (5 ng) and poly(I:C)-LNP (1 ng) for 72 h. Control groups included siCtrl- and poly(I)-LNP treatments. Cell viability was assessed by MTT assay after **A)** sequential and **B)** concurrent treatment strategies and is expressed as a percentage relative to buffer-treated cells (mean + SD, n = 3). Statistical significance is indicated as **$p \leq 0.01$ and ***$p \leq 0.001$.
